# New synthetic strategies for xanthene-dye-appended cyclodextrins

**DOI:** 10.3762/bjoc.12.53

**Published:** 2016-03-17

**Authors:** Milo Malanga, Andras Darcsi, Mihaly Balint, Gabor Benkovics, Tamas Sohajda, Szabolcs Beni

**Affiliations:** 1 CycloLab, Cyclodextrin R&D Ltd., Budapest, H-1097 Illatos út 7, Hungary; 2 Department of Pharmacognosy, Semmelweis University, H-1085 Üllői út 26, Hungary,; 3 Department of Organic Chemistry, Faculty of Science, Charles University, Hlavova 8, 12843 Prague 2, Czech Republic

**Keywords:** DMT-MM, fluorescein, rhodamine, supramolecular assembly

## Abstract

Xanthene dyes can be appended to cyclodextrins via an ester or amide bridge in order to switch the fluorescence on or off. This is made possible through the formation of nonfluorescent lactones or lactams as the fluorophore can reversibly cyclize. In this context we report a green approach for the synthesis of switchable xanthene-dye-appended cyclodextrins based on the coupling agent 4-(4,6-dimethoxy-1,3,5-triazin-2-yl)-4-methylmorpholinium chloride (DMT-MM). By using 6-monoamino-β-cyclodextrin and commercially available inexpensive dyes, we prepared rhodamine- and fluorescein-appended cyclodextrins. The compounds were characterized by NMR and IR spectroscopy and MS spectrometry, their UV–vis spectra were recorded at various pH, and their purity was determined by capillary electrophoresis. Two potential models for the supramolecular assembly of the xanthene-dye-appended cyclodextrins were developed based on the set of data collected by the extensive NMR characterization.

## Introduction

Cyclodextrins (CDs) are cyclic oligosaccharides consisting of 6, 7 or 8 glucopyranose units (α-, β- and γ-CD, respectively). Native CDs do not adsorb light in the UV–vis region (200–800 nm), but they can be converted into spectroscopically active compounds by modification with a chromophore/fluorophore unit. Fluorophore-appended CDs can be appropriate systems to detect spectroscopically inert molecules by guest-induced spectroscopic changes associated with the formation of inclusion complexes. These fluorescent CDs exhibit remarkable molecular recognition abilities, discriminating shape, bulkiness and polarity of the guests [1–2]. Furthermore, CDs can be directly modified with fluorophores for labeling in order to assess if these versatile molecules cross biological barriers (e.g., cell membrane, blood–brain barrier) and to follow their distribution in living matter [3].

**Figure 1:**
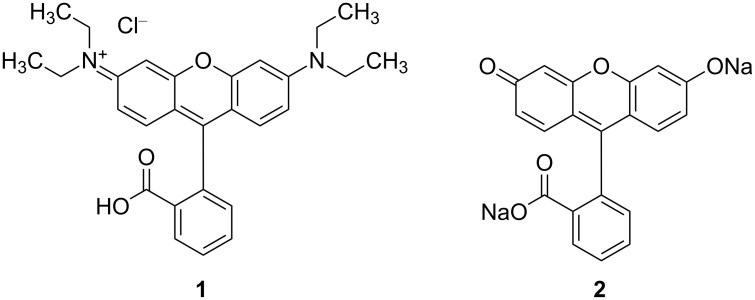
Structures of fluorescent xanthene dyes. Rhodamine B·HCl **1** and fluorescein disodium salt **2**.

Among the fluorescent dyes, the group of fluorophores based on xanthene scaffolds is one of the most popular. Two representatives of this class are fluorescein and rhodamine (Figure 1), which have been applied as chemosensors [4] and have been widely exploited in various areas such as cell biology, microscopy, biotechnology, and ophthalmology due to their versatile photophysical properties. However, the chemical modification of this evergreen class of dyes is still an ongoing process [5–6].

Fluorescein is the most widely used fluorescent probe in biological applications and in particular for covalently labeling proteins. Rhodamine derivatives are robust dyes that find application as fluorophores for microscopy, in cell sorting, in photodynamic therapy and in colorimetric enzymatic tests (ELISA).

Although it is possible to modify xanthene dyes with specific functional groups (e.g., isothiocyanates, maleimide, succinimidyl), enabling them to react with amine groups, such chemical modifications dramatically affect the cost of the dye because of the laborious purification process. In order to reduce cost, one approach is to perform the condensation reaction that leads to the formation of the xanthene dyes by using previously functionalized reagents [7]. This approach is difficult to generalize and does not involve a common synthetic plan for different xanthene dyes since their core is different. Another possibility is to modify less expensive, unfunctionalized, commercially available xanthene dyes; for instance, both rhodamine B and fluorescein are commercially available at a reasonable price. Since the presence of a carboxylic moiety is a common feature to most of the xanthene-based dyes, it is easy to develop a common synthetic plan for the activation of the dye toward biological systems based on the modification of this functional group. The most logical way for generating an active dye able to target an amine-bearing system would be the formation of an amide bond. However, in spite of the numerous attempts, the modification of this specific position still (at least in a bio-compatible environment) remains a challenge. In the case of rhodamine, the modification of the carboxylic moiety, to the best of our knowledge, is always performed in organic solvent under harsh conditions [8].

The synthesis and the use of 4-(4,6-dimethoxy-1,3,5-triazin-2-yl)-4-methylmorpholinium chloride (DMT-MM) as a coupling agent for the formation of amides and esters was first reported by Kunishima et al. [9]. This compound possesses advantageous properties as it can be prepared in gram scale from inexpensive reagents, it is stable in air, it does not adsorb water, and the water soluble co-products formed during the coupling reaction can be easily removed from the reaction crude. All these characteristics make DMT-MM a convenient tool for the synthesis of amides and esters [10].

The first example of xanthene-dye-appended cyclodextrin based on the modification of the carboxylic moiety of the fluorophore was reported by Ueno’s group [11]. In the study, they modified 6-monotosyl-β-CD with the sodium salt of fluorescein, resulting in a fluorescein β-CD derivative connected through an ester bond. The main idea of the synthetic step was to promote the nucleophilic substitution between 6-monotosyl-β-CD and the carboxylate of the fluorescein by adjusting the pH of the aqueous solution around pH 6 [12]. Some years later the same group reported on a fluorescein-modified γ-CD and its properties as a sensor and as a charge-changeable receptor for detecting organic acids [13]. The synthetic strategy was still based on the ester formation between the fluorophore and the CD scaffold, but the conditions were slightly modified. As a starting material, 6-monoiodo-γ-CD was chosen instead of the more labile 6-monotosyl-γ-CD and anhydrous DMF (under nitrogen atmosphere) substituted for the aqueous environment of the previous β-CD coupling.

The first rhodamine-modified CD was reported by Harada [14]. 6-Monoamino-α-CD was coupled with rhodamine B in the presence of *N,N'*-dicyclohexylcarbodiimide (DCC) and hydroxybenzotriazole (HOBt) in anhydrous DMF under inert atmosphere in order to connect the two moieties through an amide bond. The fluorescent CD derivative was used to observe the movement of a rotaxane immobilized on glass substrates.

Hasegawa et al. [15] were the first to develop rhodamine labeled CDs for biological purposes. They synthesized two different fluorescent β-CDs and showed their utility as new fluorogenic probes for monitoring pH of HeLa cells. The synthetic strategy was based first on the modification of the CD scaffold with two different linkers (both polyamines) and then on the coupling of the terminal amino group of the linkers with rhodamine B. The coupling conditions were slightly different from those reported by Harada [14]. The solvent was a mixture of pyridine and DMF, the activating agents for the carboxylic acid were HOBt and 1-ethyl-3-(3-dimethylaminopropyl)carbodiimide (EDC).

Fang and co-workers designed a ratiometric sensor for detecting mercury ions in aqueous media, some biological fluids and living cells based on a rhodamine-modified β-CD [16]. The synthetic strategy was a three-step procedure: rhodamine B was modified into a spirolactam rhodamine ethylenediamine, 6-monotosyl-β-CD was reacted with ethylenediamine in order to obtain the equivalent monosubstituted derivative, and finally, the coupling between the two molecules was performed. The coupling with the 6-mono(2-aminoethylamino)-β-CD was obtained by in situ conversion of the terminal amino group of the fluorophore into isothiocyanate group.

To summarize, fluorescein has been connected to CDs through an ester bond by performing the reaction in water or DMF. Although the synthetic procedure is well described, the experimental synthesis steps and the purification process are not described in enough detail. The characterization of the molecule is not exhaustive since a clear indication of the ester formation is missing. Rhodamine has been connected in organic solvents through an amide bond with DCC or EDC as coupling agents. The molecules have not been characterized in detail since only partial spectroscopic data have been reported.

In this study, we developed a general, green, and inexpensive method for the synthesis of xanthene-appended CDs. Since most of the xanthene dyes possess a carboxylic moiety (only a few examples are known that lack this moiety) we based our strategy on the formation of an amide bond. In particular, we reacted 6-monoamino-β-CD (NH_2_-β-CD) with fluorescein and rhodamine in alkaline aqueous media at room temperature in the presence of the coupling agent DMT-MM, thus producing the fluorescent derivatives. This method can not only be applied to CDs, but may represent a new general approach for the modification of xanthene dye in water, thus affording amide or ester derivatives.

When a fluorophore is used for the labeling of a single-isomer CD, the resulting molecule is rarely the fully substituted, fluorescent single-isomer CD; more commonly, the system is a mixture of unsubstituted and substituted CD derivatives. Although some data are reported in the literature for the characterization of fluorescently labeled CDs, a full NMR characterization, to the best of our knowledge, has never been presented and the spectroscopic data always refer to the mixture obtained by an incomplete fluorescent labeling and not to the fluorescent single-isomer CD derivative. The incomplete fluorescent tagging is reflected by, for example, broad NMR signals [17].

If the goal of the derivatization is the fluorescent labeling of a randomly substituted CD derivative, the situation is even more challenging. A suitable synthetic strategy for obtaining a homogeneous, fluorescent, randomly substituted CD derivative (where each compound is modified with a fluorescent moiety) is to perform the random substitution on a fluorescent single-isomer CD [18]. If the substrate for the fluorescent labeling is a randomly substituted CD derivative, most commonly, the obtained mixture will contain fluorescently labelled and unlabeled isomers.

In this work, our purpose was to develop a generic synthetic method for the derivatization of CDs with commercially available xanthene dyes and to isolate and characterize the fluorescent compounds as single isomers.

## Results and Discussion

**Figure 2:**
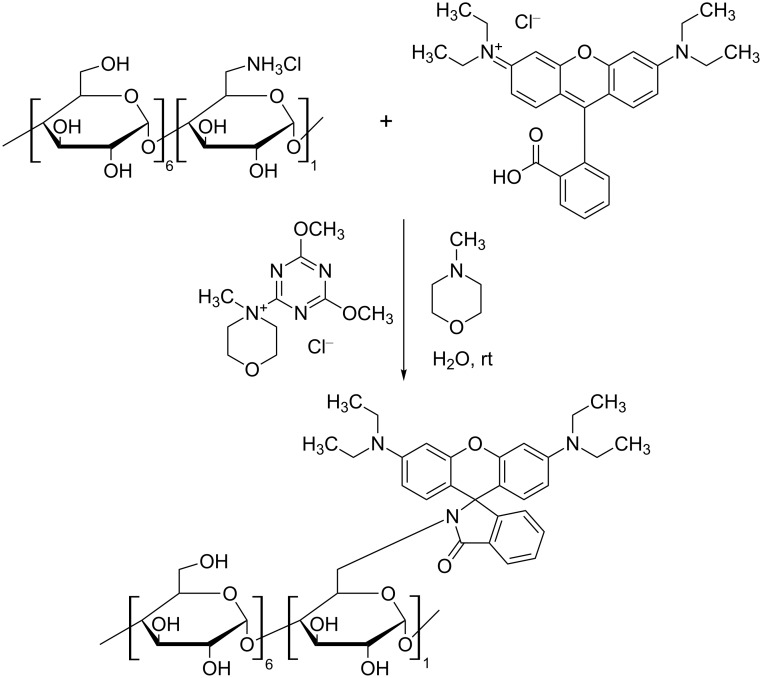
Reaction scheme for the synthesis of rhodamine-appended β-CD.

In Figure 2, the reaction scheme for the rhodamine-appended β-CD derivative (Rho-β-CD) is shown as a representative example. The synthetic strategy is based on the condensation reaction between an amine-bearing CD and the carboxyl moiety of the xanthene dye promoted by the coupling agent DMT-MM. It is accepted that the carboxylic acid has to be deprotonated in order to generate an activated ester with DMT [19]. The activated species undergoes attack by the amine, thus generating the desired amide bond.

The xanthene dyes, the β-CDs and the coupling agent are highly soluble in water, thus allowing for a homogenous reaction that occurs at room temperature. Since β-CD modified with amines is more stable in the HCl form (in order to avoid decomposition), during the reaction, an additional base such as *N*-methylmorpholine (NMM) or NaOH is required. If amine-bearing CDs are used as free bases, the additional base can be omitted. The synthesis of the reactions simply consists of the selective precipitation of the target compound with acetone and removal of the unreacted dye by filtration. The purity of the starting dye is a crucial parameter for the outcome of the reaction, since it will greatly affect the crude composition and consequently the purification process. If the purity of the starting dye is satisfactory, selective precipitation/filtration are the only steps required for obtaining a fluorescent-appended β-CD derivative of acceptable purity (>90%, based on thin-layer chromatography (TLC)). If the starting fluorophore is a mixture of fluorescent (but not clearly identified) dyes, then chromatography is needed to obtain the desired purity.

### Rhodamine-appended β-CD

**Figure 3:**
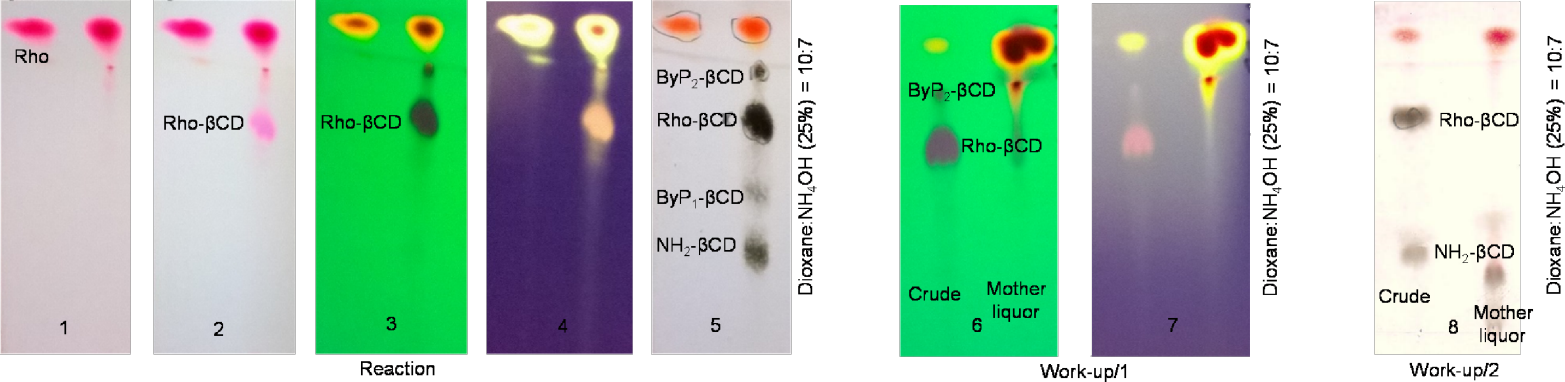
TLC plates at different development stages for monitoring the composition of Rho-β-CD crude (left panel) and TLC for evaluating the effectiveness of the work-up (right panels).

Rhodamine B in HCl form (Rho·HCl) was sourced in good purity, allowing for a clean formation of the product with a very low amount of rhodamine-related byproducts (not detectable by TLC), a minute amount of two β-CD-related byproducts (see ByP_1_-, ByP_2_-β-CD in TLCs in Figure 3, the sum of the two being less than 5% based on the intensity of the spots on TLC-5) and some unreacted NH_2_-β-CD (less than 5% based on the intensity of the spots on TLC-5). The reaction was completed in a couple of hours, at room temperature, in an aqueous environment. TLC provides an unambiguous identification of the product, and the behavior of the product in the selected eluent gives structural indication about the possible prototropic form assumed from the dye in the conjugate.

The left panel of Figure 3 shows the TLC plates used for monitoring the composition of Rho-β-CD crude at different development stages, and in particular, immediately after the removal from the developing chamber (1), after heating (2), under UV excitation at 254 nm (3), under UV excitation at 366 nm (4), and finally, after charring (5). The right panels in Figure 3 show the effectiveness of the selective precipitation/filtration during the work-up and in particular it reveals that the unreacted dye can be separated from the product (TLC-6, TLC-7 under UV excitation at 254 nm and at 366 nm, respectively) while the unreacted NH_2_-β-CD and the nonfluorescent ByP_1_-β-CD are more challenging to remove (TLC-8, after charring).

In the left panel of Figure 3, the unreacted dye (Rho) is clearly detectable at any stage of the development, while the product (Rho-β-CD) is detectable only after heating the TLC plate and appears as a slight pink spot (TLC-2 in Figure 3). This spot is colored, strongly UV active, nonfluorescent and charrable (TLC-2, -3, -4 and -5, respectively in Figure 3). The presence of color and the charrability indicates that the compound contains both the chromophore and the CD scaffold. The strong UV activity and the nonfluorescence suggest that the chromophore is prevalently in lactam form [20]. As it is also known that β-CD has the ability to preferably complex the cyclic form of rhodamine B [21], complexation may play a role in the stabilization of the lactam form of the rhodamine-appended CD derivative. All this information taken together confirms the presence of the product/conjugate and add to the structural elucidation on the system.

Concerning the synthesis, the first part consists of the removal of the unreacted dye with acetone. As shown in Figure 3, after selective precipitation/filtration with acetone, most of the unreacted dye and ByP_2_-β-CD remain in the mother liquor (TLC-6, TLC-7 in Figure 3). At this stage, the crude already has acceptable purity (>90% based on TLC), but flash chromatography with a CH_3_CN–H_2_O gradient elution permits the removal of the remaining CD-related byproducts and further increases the purity. After this additional purification step, the compound, Rho-β-CD, has been extensively characterized by NMR spectroscopy.

### NMR characterization of Rho-β-CD

**Figure 4:**
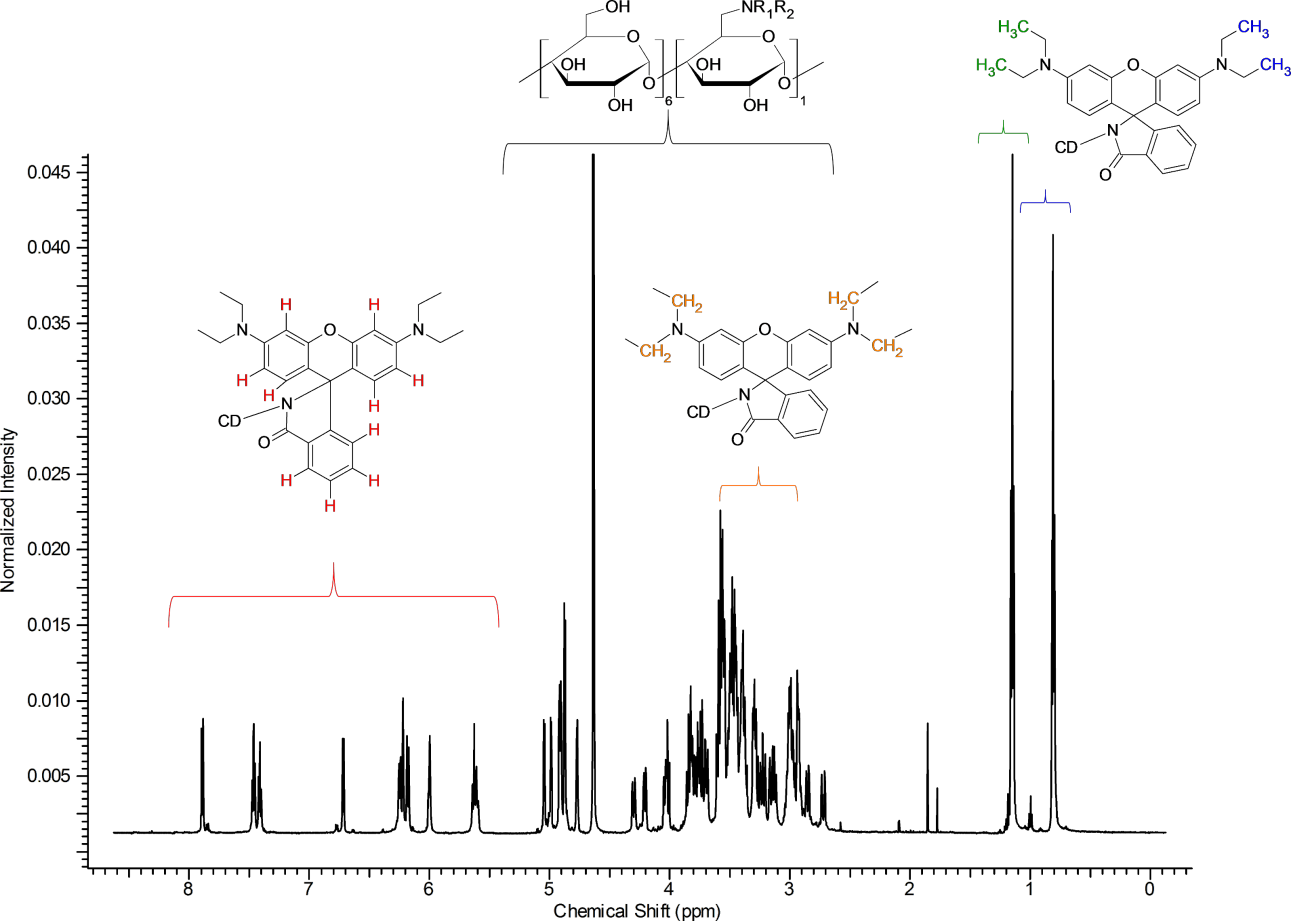
^1^H NMR spectrum of Rho-β-CD with partial assignments (D_2_O, 500 MHz, 298 K).

The proton NMR spectrum shown in Figure 4 is a typical spectrum of an asymmetric cyclodextrin. The sharpness of the peaks suggests the high purity of the compound.

The two constituent parts of the molecule can be easily recognized in Figure 4. The resonances in the aromatic (5.5–8.0 ppm) and aliphatic (0.5–1.5 ppm) regions belong to the rhodamine moiety, while those observed between 2.5–5.5 ppm belong to the CDs and also include the methylene moieties of the fluorophore. The integration of the signals perfectly fits the theoretical values for a monosubstituted rhodamine-β-CD derivative. This is also confirmed by the found value of the pseudo-molecular ion during the electrospray ionization mass spectrometry (ESIMS) analysis (see Experimental part).

The resonance signals in the aromatic region are well resolved (see Supporting Information File 1, Figure S1) and the cross-linking with the data obtained by the COSY and DEPT-ed-HSQC spectra (see Supporting Information File 1, Figures S2 and S3, respectively) allowed for the complete assignment (the data are in agreement with the literature [22]). The multiplicity of the signals can be clearly determined. This is not an obvious characteristic for rhodamine-based CD derivatives since the aromatic signals of these compounds are usually represented by very broad signals. The presence of broad signals is characteristic for randomly substituted compounds as the NMR signals are made from the frequencies of all the compounds composing the mixture. An incomplete fluorescent tagging of the starting single-isomer β-CD (and/or inadequate purification), as well as aggregation of the compound, can cause broadening of the peaks [23].

In the proton spectrum in Figure 4, the good resolution in the anomeric region can be appreciated; several doublets can be clearly identified (see Supporting Information File 1, Figure S4). The separation of the anomeric doublets is a fundamental requisite for the complete assignment of an asymmetric molecule [24]. The core of the CD region between 2.5–5.5 ppm (Figure 4) is rather crowded and cannot be elucidated without the use of 2D techniques. On the other hand, the two signals in the aliphatic region can be easily assigned to the alkyl side chains of the rhodamine. Another important set of information that can be deduced/extrapolated from the careful analysis of the spectrum in Figure 4 concerns the asymmetry of the molecule. The proton spectrum of the rhodamine B both in lactone (Supporting Information File 1, Figure S5) and HCl form (Supporting Information File 1, Figure S6) shows only one kind of signal for the alkyl groups (CH_3_ and CH_2_ in Supporting Information File 1, Figures S5 and S6) and for the aromatic protons of the xanthene moiety (protons E, F, G in Supporting Information File 1, Figures S5 and S6). This means that the differentiation in multiple NMR signals in the Rho-β-CD conjugate is strictly related to the presence of the CD scaffold. Because of the chirality of the cyclodextrin part, the two phenyl rings of the xanthene moiety are formally diastereotopic, and consequently, anisochronous even without any complexation. This fact can be proven by recording the ^1^H NMR spectrum of the Rho-β-CD in deuterated DMSO, a solvent known to dissociate inclusion complexes (Supporting Information File 1, Figure S7). The presence of two kinds of signals for the methyl units (at 1.06 ppm and 1.14 ppm) in the spectrum recorded in deuterated DMSO confirms the aforementioned observation.

The analysis of the DEPT-ed-HSQC spectrum (see Supporting Information File 1, Figure S8 for the full spectrum) gives further information on the product. The compound is unambiguously substituted on the primary side.

**Figure 5:**
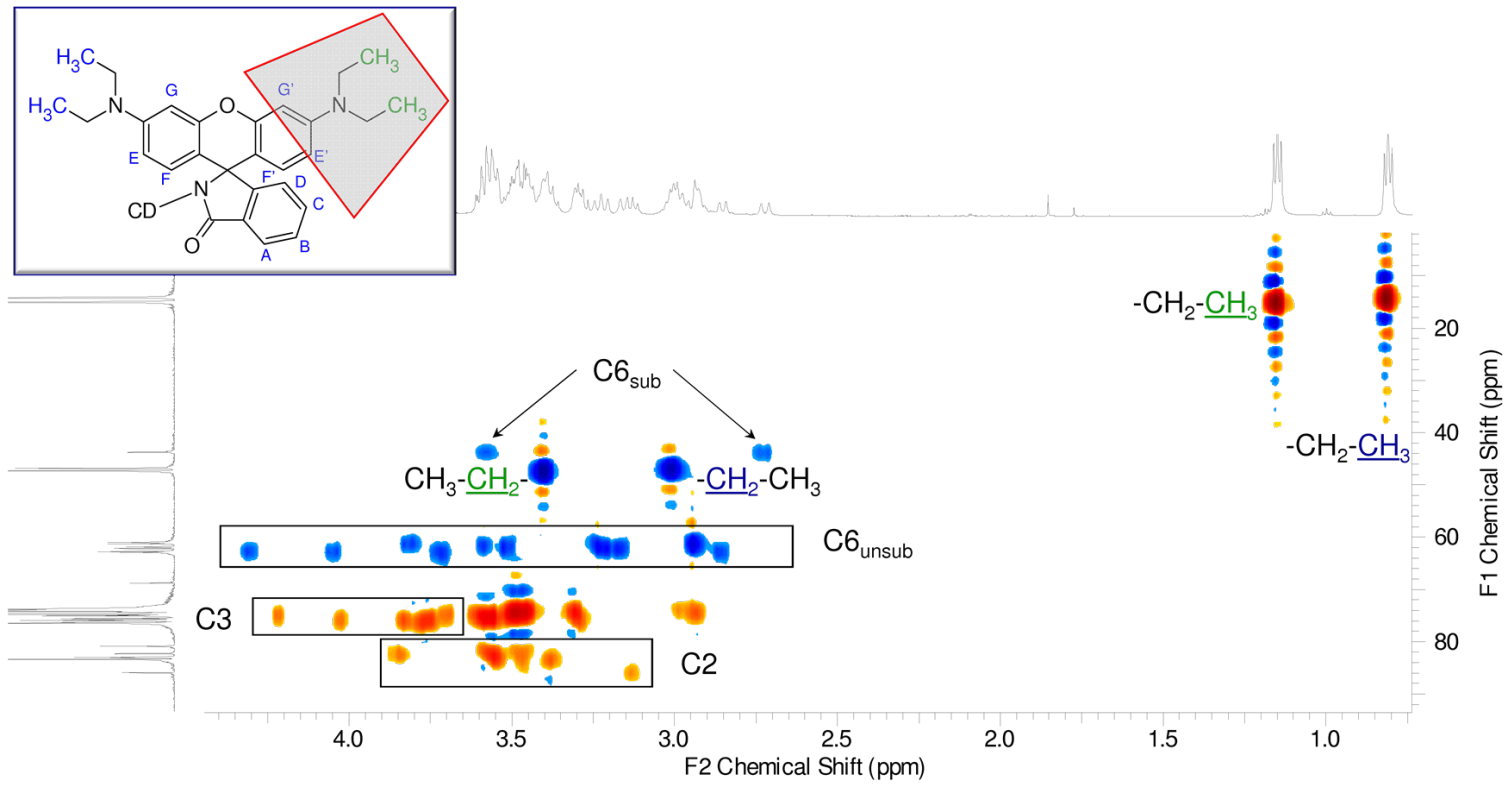
Expansion of DEPT-ed-HSQC spectrum of Rho-β-CD with partial assignments (D_2_O, 500 MHz, 298 K).

The frequencies at around 40 ppm (C6_sub_ in Figure 5) correspond to the methylene moiety of the glucose unit that bears the fluorophore. The two frequencies have similar carbon signals, but different protons since the two protons of the methylene unit are not magnetically equivalent (C6_sub_ in Figure 5). The signals of the anomeric region (Supporting Information File 1, Figure S8) are very diffuse as those of the CD core. It is also possible to distinguish the two signals of the methylene units of the side chains of the rhodamine (around 47 ppm) from the unsubstituted methylene moiety of the glucose units (C6_unsub_ in Figure 5). The assignment of the frequencies of the alkyl chains of the fluorophore is based on the 2D total correlation spectroscopy (TOCSY) spectrum (Supporting Information File 1, Figure S9), while the partial assignments of the C2 and C3 units (Figure 5) are based on a combination of correlation spectroscopy (COSY) (Supporting Information File 1, Figure S2), DEPT-ed-HSQC (Supporting Information File 1, Figure S8) and TOCSY (Supporting Information File 1, Figure S9).

In the aromatic region of the DEPT-ed-HSQC spectrum (see Supporting Information File 1, Figure S3) of Rho-β-CD, all ten of the aromatic protons of the fluorophore can be clearly distinguished.

The carbon spectrum shows very sharp peaks (Supporting Information File 1, Figure S10). The presence of a single carbon signal (around 172 ppm) in the region of the carboxylic amide unambiguously proves the effectivity of the coupling as well as the purity of the compound.

**Figure 6:**
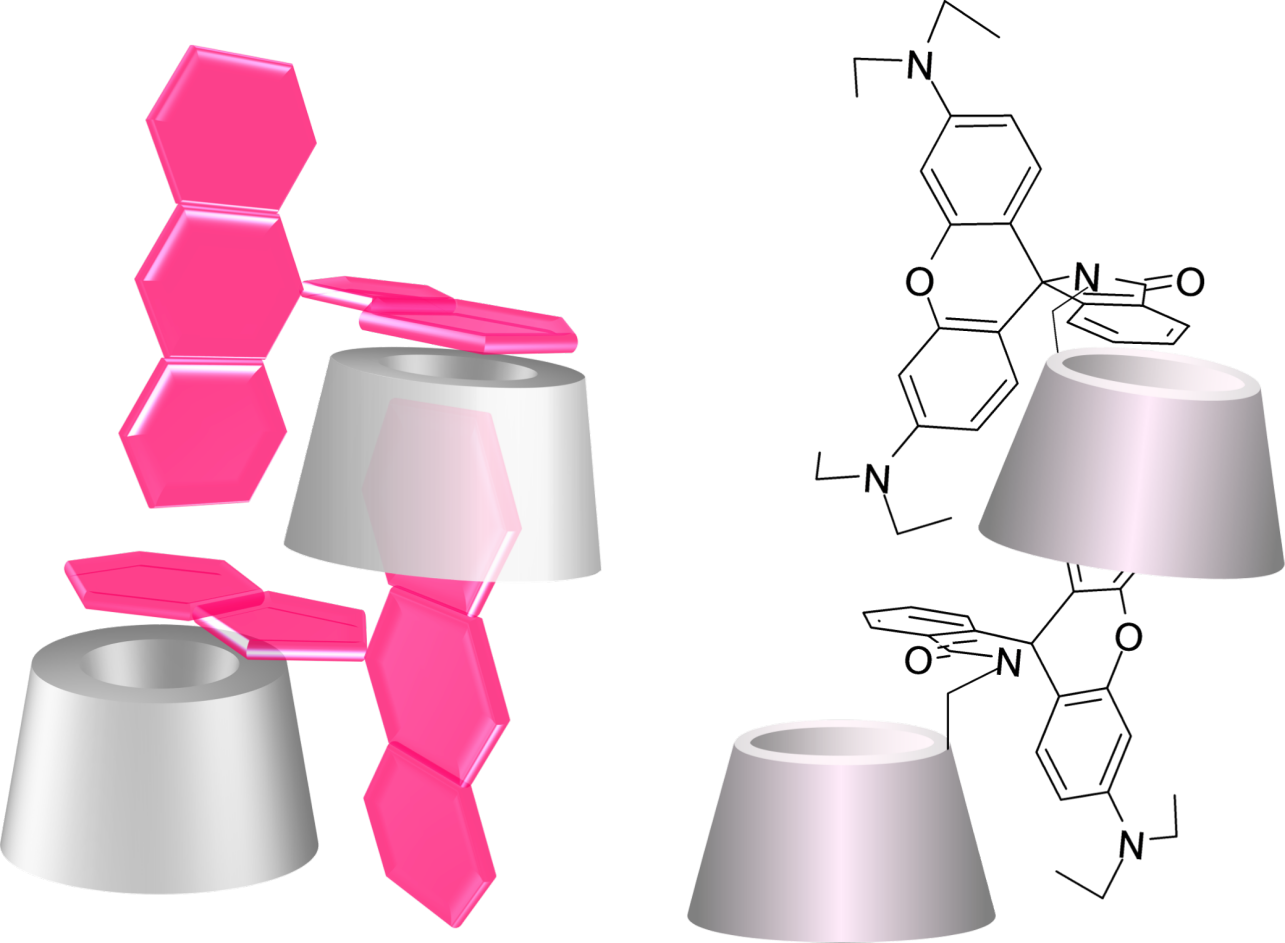
Cartoon models for the possible intermolecular inclusion mode of Rho-β-CD in solution (3D perspective view on the left and 3D structural model on the right).

The cross-linked analysis of the 2D rotating frame nuclear Overhauser effect spectroscopy (ROESY) spectrum (Supporting Information File 1, Figures S11, S12 and S13) and the DEPT-ed-HSQC spectrum (Supporting Information File 1, Figure S14) generates a set of information useful for the determination of the spatial position of the fluorophore with respect to the CD scaffolds and for the analysis of the preferred conformation assumed by Rho-β-CD in solution. These data, together with the results presented by Wang et al. [25] about the crystal structure of rhodamine B in lactone form, allowed us to propose the model shown in Figure 6 for the intermolecular inclusion mode of Rho-β-CD. This model for the supramolecular assembly is also in agreement with previous data about the inclusion complexation of organic dyes with β-CD dimers [26].

In more detail, the analysis of the 2D ROESY spectrum reveals that the methyl units of the rhodamine side chains that resonate at 0.81 ppm (the side chains that are “uncomplexed”) show cross peaks to 3.02 ppm (Supporting Information File 1, Figure S12), 5.63 ppm and 6.02 ppm (Supporting Information File 1, Figure S13). These three cross peaks are the result of intramolecular interactions between the methyl units and the adjacent methylene units of the rhodamine side chains (3.02 ppm) and between the methyl units and the protons G (5.63 ppm) and E (6.02 ppm) of the xanthene units (Supporting Information File 1, Figure S3), respectively. The two additional cross peaks at 7.91 and 6.74 ppm are the result of the spatial interactions of the methyl units with the protons A and D of the aromatic ring adjacent to the spirolactam unit (Supporting Information File 1, Figure S3). Assuming that the spirolactam is positioned perpendicular to the plane described by the xanthene unit (as reported in [25]), then the aforementioned, two additional cross peaks are the result of intermolecular interactions (see models in Figure 6).

The methyl signals at 1.15 ppm (the side chains that are “complexed”) show several cross peaks indicating the diffused spatial interactions between the side chains of the fluorophore and the lower part of the cavity (carbons 3) of the CD scaffold (cross peaks at 3.57, 3.77, 3.83, 4.03 ppm in Supporting Information File 1, Figure S12). The same methyl moieties also interact with the protons G’ and E’ (cross peaks at 6.24 ppm in Supporting Information File 1, Figure S12 and cross peak at 6.27 ppm in Supporting Information File 1, Figure S13) of the xanthene units (Supporting Information File 1, Figure S13). The first set of cross peaks (at 3.57, 3.77, 3.83, 4.03 ppm) must be the result of intermolecular interactions between fluorophore units and a close CD scaffold since the xanthene moieties show exclusively cross peaks with the lower part of CD cavity. If a self-inclusion scenario would occur then one would expect a set of cross peaks between the xanthene moiety, the primary side of the CD (carbons 6) and the upper part of the cavity of the CD (carbons 5); however, this was not detected in our case. The second set of cross peaks (at 6.24 and 6.27 ppm in Supporting Information File 1, Figures S12 and S13, respectively) is the result of intramolecular forces due to the spatial proximity of the methyl units to the adjacent aromatic ring of the xanthene unit.

It is also worth noting that the protons A and D of the aromatic ring adjacent to the spirolactam unit (Supporting Information File 1, Figure S3) at 7.91 and 6.74 ppm show cross peaks at 3.23 and 3.20 ppm (Supporting Information File 1, Figure S13), respectively. These two frequencies correspond to the protons of unsubstituted methylene groups of the CD unit (Figure 5 and Supporting Information File 1, Figure S14). These intramolecular cross peaks are possible only if the plane of the aromatic ring adjacent to the spirolactam ring is set parallel to the primary rim of the CD.

Finally, the aromatic protons that resonate at 6.19, 6.24, and 6.27 ppm (protons F’, G’, E’ in Supporting Information File 1, Figure S3) show cross peaks at 3.3, 3.46, 3.77, 4.03 and 4.23 ppm as reported in Supporting Information File 1, Figures S12 and S13. These last frequencies correspond to some of the protons of the lower part of a CD cavity (carbons 3), suggesting an intermolecular, partial complexation of the xanthene moiety into a close CD cavity.

To summarize, the compound Rho-β-CD was obtained in high purity and was thoroughly investigated by NMR spectroscopy. The collected data unambiguously proved that the compound is monosubstituted on the primary side. Morover, the analysis of the NMR spectra revealed that the fluorophore is partially complexed. The additional data obtained by the analysis of the ROESY spectrum resulted in the proposed model (Figure 6) for the intermolecular inclusion mode of the compound.

### IR and UV–vis characterization of Rho-β-CD

In Supporting Information File 1, Figure S15 the IR spectra of Rho-β-CD, rhodamine B in acidic form (Rho∙HCl) and rhodamine B in lactone form (Rho-B lactone) are shown. The analysis of the spectra unambiguously proved that the fluorophore in Rho-β-CD is in lactam form. The frequency at 1755.9 cm^−1^ in the IR spectrum of Rho-B lactone belongs to the carbonyl stretching of the γ-lactone moiety of the dye and this value is very similar to that found in the carbonyl region of the IR spectrum of Rho-β-CD at 1755.4 cm^−1^. This last frequency is typical for the carbonyl stretching of a γ-lactam ring [27], thus demonstrating that the fluorophore in Rho-β-CD is (if isolated according to the procedure reported in the Experimental section) in a nonfluorescent cyclic form.

The same conclusion can be also reached through the analysis of the UV–vis spectra (Supporting Information File 1, Figure S16). The UV–vis spectra of Rho-β-CD vary according to the pH of the solution in a different manner compared to the free rhodamine B. The free fluorophore shows a strong absorbance at around 550 nm and maintains its fluorescence at any of the examined pH (see Experimental section for details). In the case of Rho-β-CD, this absorption maximum undergoes a hypochromic shift at alkaline and neutral pH, and under these conditions, the compound is not fluorescent. For the xanthene-appended derivative, the UV maximum at around 550 nm only appears at pH 3 (the absorbance increases with time) and under these solution conditions, Rho-β-CD is a fluorescent compound. The phenomenon can be easily explained on the basis of the change of conformation of the fluorophore according to the pH. The opening of the lactam ring is catalyzed by acidic conditions, thus switching the equilibrium of the prototropic forms of the dye towards the fluorescent amide form [28].

### Fluorescein-appended β-CD

Fluorescein disodium salt (Flu-Na) was purchased in high purity allowing the formation of the product, but with moderate yield. For this reaction, several fluorescein-related byproducts could be detected by TLC (Supporting Information File 1, Figure S17) and the starting NH_2_-β-CD·HCl could be only partially converted to Flu-β-CD (20–30% conversion based on TLC). Although different attempts were made in order to enhance the conversion (such as tuning the pH of the reaction, using NH_2_-β-CD as free base as starting material, replacing the base NMM with NaOH and reacting the lactone form of the fluorophore instead of the sodium salt), the improvements were not substantial. The reason for the partial conversion under the selected alkaline conditions can be related to the appearance of several dye-related byproducts (ByP_1_-, ByP_2_-, ByP_3_-Flu in TLC in Supporting Information File 1, Figure S17). Under the selected aqueous alkaline conditions, the phenol moiety of the fluorescein is mainly deprotonated and as phenolate can participate in the formation of fluorescein-based acyl derivatives (such as esters or anhydride, see Supporting Information File 1, Figure S17). The formation of these byproducts could lead to depletion of the coupling agent by conversion to 2-hydroxy-4,6-dimethoxy-1,3,5-triazine (DMM-OH) and could explaining the moderate conversion of the starting material. It is worth emphasizing that the coupling of fluorescein based on DCC/HOBt in organic solvents (as described in [14] for example) generates even more complicated mixtures. As shown in the TLC in Supporting Information File 1, Figure S14, an additional, nonfluorescent, unidentified, CD-related byproduct (ByP_1_-CD) can be clearly detected in the crude and the amount of this byproduct is rather significant. As a consequence, the aqueous method based on DMT-MM is more favorable in terms of the amount of the product in the crude mixture and in terms of the purification of the crude composition.

The removal of the unreacted dye as well as the dye-related byproducts can be achieved by selective precipitation/filtration with acetone. Flash chromatography using a 10:5:1 (v/v/v) CH_3_CN/H_2_O/NH_4_OH (25%) ratio as eluent permits the removal of the unreacted NH_2_-CD-related impurities. At this stage the compound, Flu-β-CD, has been extensively characterized by spectroscopic techniques.

### UV–vis characterization of Flu-β-CD

In Supporting Information File 1, Figure S18, the UV–vis spectra of fluorescein disodium salt and of Flu-β-CD are shown. Under acidic conditions (at pH 3), the UV–vis spectrum and the fluorescence of the free dye changes remarkably. In particular, the UV band at around 490 nm is almost completely suppressed and the fluorescence, under irradiation at 366 nm, decreases substantially. This behavior is in agreement with the formation of the nonfluorescent lactone form under acidic conditions [12]. The lactone formation is mainly responsible for the fluorescence quenching of the dye. The UV–vis spectra of Flu-β-CD resemble those of fluorescein, and the fluorescence of the compound is heavily quenched at pH 3. By taking into consideration all these data, one can assume that the fluorescein moiety of Flu-β-CD exists mainly in the open amide form (at neutral and alkaline pH) and that the compound acts as a complementary molecular switch to Rho-β-CD. While Rho-β-CD exhibits fluorescence at acidic pH, Flu-β-CD shows fluorescence at both neutral and alkaline pH.

### NMR characterization of Flu-β-CD

**Figure 7:**
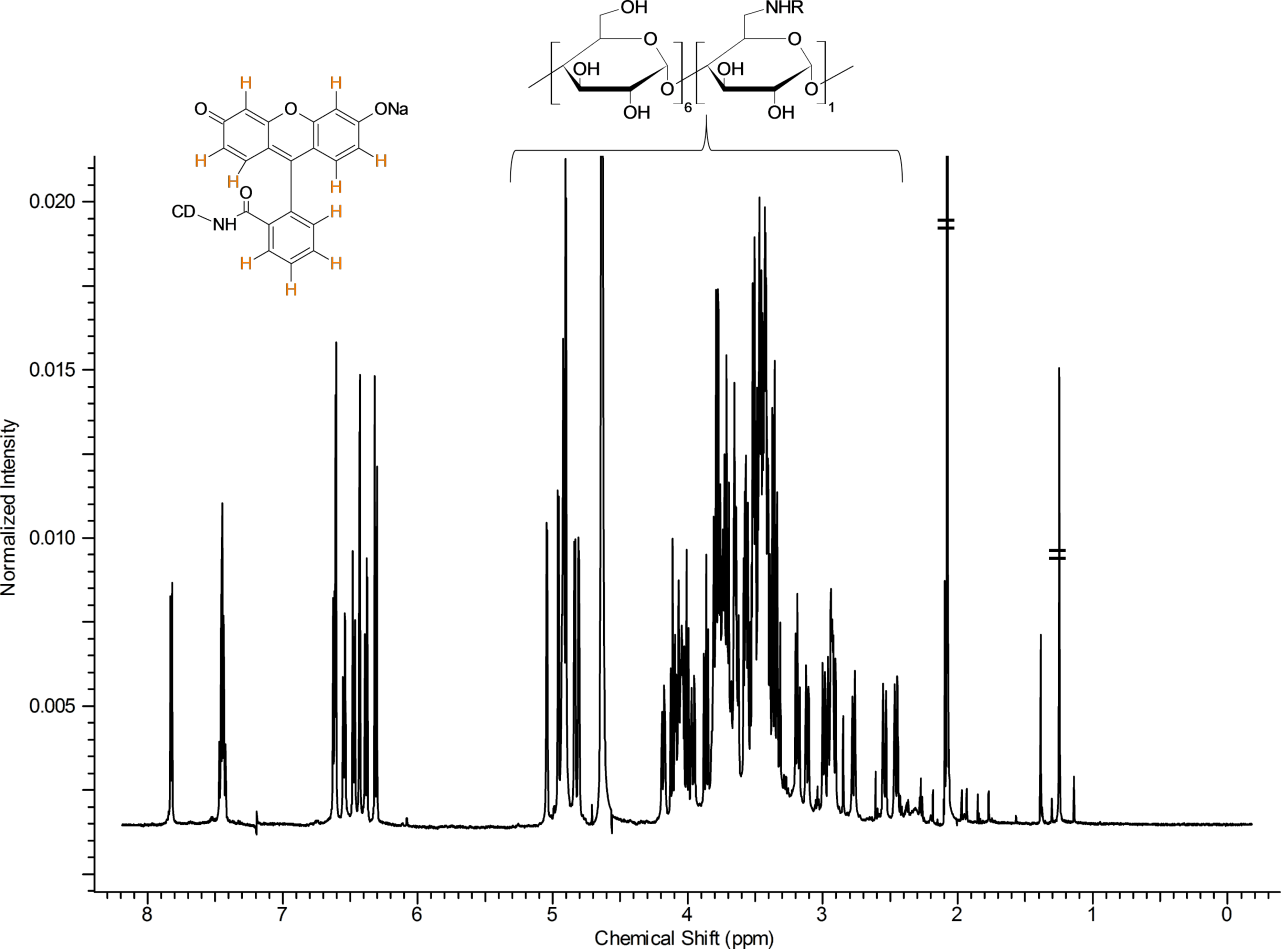
^1^H NMR spectrum of Flu-β-CD with partial assignments (D_2_O, 500 MHz, 298 K).

The ^1^H NMR spectrum shown in Figure 7 is a typical spectrum of an asymmetric cyclodextrin. The sharp and well-resolved signal suggests the high purity of the compound.

The two constituent parts of the molecule can be easily recognized in Figure 7. The signals in the aromatic region (between 6.0–8.0 ppm) belong to the fluorescein moiety, while the set of signals between 2.2–5.5 ppm belongs to the CD. The integration of the signals corresponds to the theoretical values for a monosubstituted, fluorescein-β-CD derivative, as also confirmed by the value of the pseudo-molecular ion found during the ESIMS analysis (see Experimental section).

The signals in the aromatic regions are well resolved (Supporting Information File 1, Figure S19) and the analysis of H–H *J* coupling and the cross-linking with the data obtained by the COSY and DEPT-ed HSQC spectra (Supporting Information File 1, Figures S21, S22 and Supporting Information File 1, Figure S24, respectively) allowed for a complete assignment of the resonance frequencies. It is worth noting that all ten of the aromatic protons of the fluorophore can be identified both in DEPT-ed-HSQC and ^1^H NMR spectra. The possibility to resolve all the aromatic protons of the dye in the ^1^H NMR spectrum (Supporting Information File 1, Figure S19) is related to the asymmetry of Flu-β-CD. In the case of the free fluorescein sodium salt, the resolution of the proton couples H7/H8, H6/H9, H5/H10 is not possible without the addition of a resolving agent (Supporting Information File 1, Figure S20).

In the 6.0–8.0 ppm region of the ^1^H NMR spectrum of Flu-β-CD (Supporting Information File 1, Figure S19), two different kinds of aromatic coupling can be detected. The different values of the ^3^*J*_H-H_ and ^4^*J*_H-H_ couplings permit the unambiguous assignment of the coupled protons H5/H7 and H8/H10. In the proton spectrum in Figure 7, the good resolution of the anomeric region can be also appreciated; several doublets can be clearly identified. The core of the CD region between 2.2–5.5 ppm (Figure 7) is rather crowded and cannot be easily elucidated without the use of 2D techniques.

The analysis of the DEPT-ed-HSQC spectrum (Supporting Information File 1, Figure S23 for the full spectrum) gives further information on the product. The compound is unambiguously substituted on the primary side.

The frequencies at around 44 ppm (C6_sub_ in Supporting Information File 1, Figure S25) belong to the methylene moiety of the glucose unit that bears the fluorophore. The two frequencies have similar carbon signals, but different protons since the two protons of the methylene unit are not magnetically equivalent (C6_sub_ in Supporting Information File 1, Figure S25). Further convincing proof that the compound is exclusively substituted on the primary side arises from the analysis of the HMBC spectrum of the compound (Supporting Information File 1, Figure S27). In the 2D spectrum shown in Supporting Information File 1, Figure S27, the cross peak between the carbon of the carboxamide at around 174 ppm and the proton of the methylene moiety of the glucose unit that bears the fluorophore (C6_sub_) at 2.92 ppm unambiguously confirms the structure of the compound.

The signals of the CD core and the partial assignments of the C2, C3 and C4 (Supporting Information File 1, Figure S25) are based on a combination of COSY (Supporting Information File 1, Figures S21, S22), DEPT-ed-HSQC (Supporting Information File 1, Figure S23), HMBC (Supporting Information File 1, Figures S28, S29) and TOCSY (Supporting Information File 1, Figure S26) spectra.

The carbon spectrum shows sharp resonances (Supporting Information File 1, Figure S30). The presence of a single carbon signal (around 174 ppm) in the region of the carboxylic amide proves the effectivity of the coupling and the purity of the compound as well.

**Figure 8:**
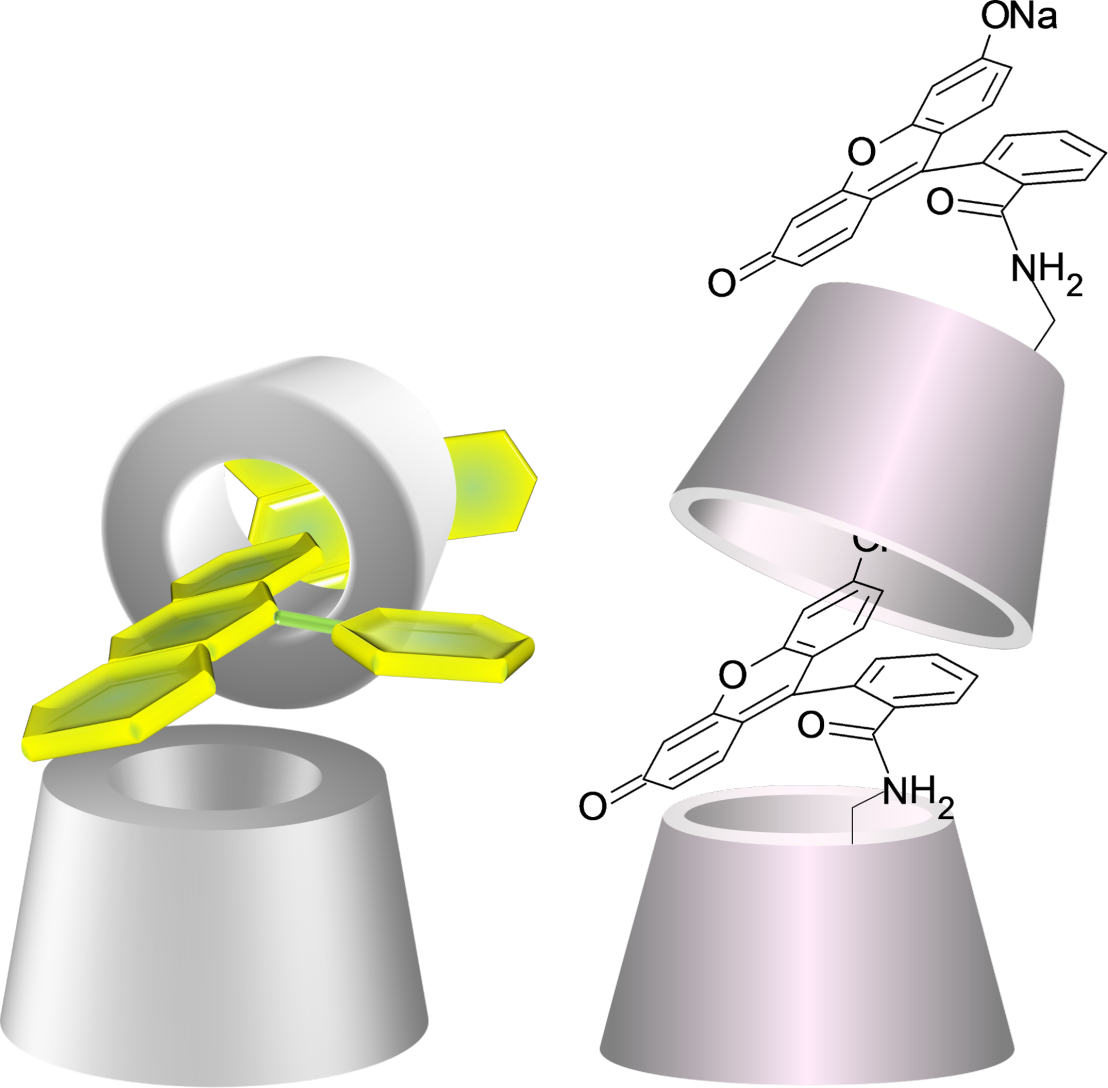
Cartoon models for the possible intermolecular inclusion mode of Flu-β-CD in solution (3D perspective view on the left and 3D structural model on the right).

The cross-linked analysis of the 2D ROESY spectrum (Supporting Information File 1, Figures S31, S32, S33) and the DEPT-ed-HSQC spectrum (Supporting Information File 1, Figures S24, S25, S34, S35 and S36) generate a set of information useful for the determination of the spatial position of the fluorophore with respect to the CD scaffolds and for the analysis of the preferred conformation assumed by the Flu-β-CD in solution. These data altogether allowed us to propose the model shown in Figure 8 for the intermolecular inclusion mode of Flu-β-CD.

In more detail, the analysis of the 2D ROESY spectrum reveals that the aromatic proton at 6.63 ppm (H4 in Supporting Information File 1, Figure S24) shows cross peaks at 3.38 ppm (Supporting Information File 1, Figure S32). This frequency corresponds to a proton of an unsubstituted methylene unit on the primary side of the CD (Supporting Information File 1, Figure S34). Since protons H1, H2 and H3 (Supporting Information File 1, Figure S24) do not show cross peaks with frequencies of the CD unit, it seems reasonable to assume that while H4 is located in proximity of the primary rim, the remaining protons of the aromatic ring are positioned externally to the primary rim and far enough not to interact with the protons of the methylene units (Figure 8).

The aromatic protons at 6.61 and 6.55 ppm (H7 and H6, respectively, in Supporting Information File 1, Figure S24) show several cross peaks as a consequence of the diffuse interaction with a CD cavity. In particular, H7 shows cross peaks at 4.08, 4.01 and 2.94 ppm, and H6 at 3.86, 3.79, 3.46 and 2.98 ppm (Supporting Information File 1, Figures S32, S33). The first set of these frequencies corresponds to different C3 (Supporting Information File 1, Figures S25, S34, S35, S36) while the second one (with the only exception at 3.36 ppm that cannot be unambiguously assigned) corresponds to protons located inside the cavity (mostly C3). These findings prove that the aromatic protons H7 and H6 interact extensively with the lower part of a CD cavity.

The aromatic proton at 6.48 ppm (H5 in Supporting Information File 1, Figure S24) shows cross peaks at 3.46, 3.35 and 2.98 ppm (Supporting Information File 1, Figures S32, S33); these frequencies correspond to protons located inside the cavity of a CD ring (Supporting Information File 1, Figures S34, S35, S36). As a consequence, the ring of the xanthene moiety that includes H5/H6/H7 interacts with the lower part of a CD cavity. Since the fluorophore is connected through the primary side of the CD, it is reasonable to assume that this set of interactions is the result of an intermolecular partial complexation between the fluorophore of a specific molecule and the lower part of the cavity of a second CD unit.

The last set of cross peaks in the aromatic region of the ROESY spectra in Supporting Information File 1, Figures S32, S33 is the result of the interactions between the aromatic proton at 6.44 ppm (H8 in Supporting Information File 1, Figure S24) and the frequencies at 4.17, 4.05, 3.65 and 3.32 ppm (Supporting Information File 1, Figures S32, S33). These frequencies correspond to protons of unsubstituted methylene units on the primary side of a CD (Supporting Information File 1, Figures S34, S35, S36). Since protons H9 and H10 (Supporting Information File 1, Figure S24) do not show cross peaks with any frequencies of the CD units, one can conclude that the xanthene moiety of the fluorophore is not placed in a parallel way to the primary rim of the CD. The plane of the xanthene moiety of the fluorophore should be twisted above the primary side of the CD in a way that H8 would be the only point of (intramolecular) interaction with the upper rim of the CD cavity.

To summarize, the compound Flu-β-CD was obtained in high purity and was thoroughly investigated by NMR spectroscopy. The collected data unambiguously proved that the compound is monosubstituted on the primary side. The analysis of the NMR spectra revealed as well that the fluorophore is partially complexed. The additional data obtained by the analysis of the ROESY spectrum resulted in the proposed model (shown in Figure 8) for the intermolecular inclusion mode of the compound.

## Conclusion

A novel green synthetic strategy for obtaining single isomer, xanthene-appended cyclodextrin was developed. The synthetic approach is based on commercially available fluorescent dyes and coupling agents, thus having clear potential for multigram scale-up. The mild, aqueous conditions, as well as the simplicity of the synthesis, make these reactions attractive tools for the preparation of fluorescent cyclodextrins connected through an amide bond.

The obtained products were isolated in high purity and extensively characterized by spectroscopic techniques. The in-depth analysis of the collected sets of NMR data resulted in the proposed models for the supramolecular interactions shown by the compounds.

The isolated molecules can be applied in the fields of chemosensing and bioimaging, while the mechanism behind the formation of the versatile, supramolecular assemblies deserves further investigation.

## Supporting Information

File 1 Experimental section, including IR and NMR spectra of the synthesized compounds.
