# Supplementary material for: New synthetic strategies for xanthene-dye-appended cyclodextrins
Source: Beilstein J Org Chem. 2016 Mar 17;12:537–48. doi: 10.3762/bjoc.12.53 (PMC4902026; doi:10.3762/bjoc.12.53)

Supporting Information

for

New synthetic strategies for xanthene-dye-appended cyclodextrins

Milo Malanga[1*], Andras Darcsi[2], Mihaly Balint[1], Gabor Benkovics[1,3], Szabolcs Beni[2], and Tamas Sohajda[1]

Address: [1]CycloLab, Cyclodextrin R&D Ltd., Budapest, H-1097 Illatos út 7, Hungary, [2]Department of Pharmacognosy, Semmelweis University, H-1085 Üllői út 26, Hungary, and [3]Department of Organic Chemistry, Faculty of Science, Charles University, Hlavova 8, 12843 Prague 2, Czech Republic

Email: Milo Malanga* - malanga@cyclolab.hu

* Corresponding author

Experimental section, including IR and NMR spectra of the synthesized compounds

Experimental

Material and methods

6-Monodeoxy-6-monoamino-β-CD hydrochloride is a fine chemical product of CycloLab. Rhodamine B HCl salt (Rho-B·HCl, ≥95%), rhodamine B base (Rho-B Lactone, dye content >97%), fluorescein disodium salt (Flu-Na, 98.5-100.5%), 4-(4,6-dimethoxy-1,3,5-triazin-2-yl)-4-methylmorpholinium chloride (DMT-MM, >96%), 4-methylmorpholine (NMM, 99%), *N*,*N′*-dicyclohexylcarbodiimide (DCC, 99%) and 1-hydroxybenzotriazole hydrate (HOBt, ≥99%) were purchased from Sigma-Aldrich. All the reagents were used without further purification. Solvents were dried by conventional methods and distilled immediately prior to use.

Silica gel coated aluminum sheets were from Merck (Art. No.: 1.05554). Plates were developed in a saturated chamber in a 1,4-dioxan:ammonium hydroxide (25%) = 10:7 (v/v), or acetonitrile/water/ammonium hydroxide (25%) = 10:5:1 (v/v/v). Visualization was achieved under UV light at 254/366 nm and by charring with a solution of EtOH (96%)/H_2SO_4 (96%) = 9:1 (v/v) by heating at 105–110 °C.

Silica gel 60 (0.063–0.200 mm) was from Merck and was used for chromatographic purification.

Analysis of TLC plates was performed with the software JustQuantify Free.

^{1}H-, ^{13}C- NMR spectra and DEPT-ed-HSQC, HMBC, TOCSY, COSY and ROESY spectra were recorded in D_2O or $(CD_3)_2SO$ (10 mg dissolved in 0.8 mL of deuterated solvent) on a Varian VXR-600 at 600 MHz at 298 K.

UV/Vis absorption spectra were recorded with a Hewlett Packard 8452A spectrophotometer.

CE experiments were conducted on an Agilent 7100 Capillary Electrophoresis System equipped with Diode Array Detector (Waldbronn, Germany).

The total fluorescent dye content was evaluated by UV-Vis spectroscopy. The estimation of the chromophore was performed based on a calibration curve using as standard the starting fluorescent dye (rhodamine B or fluorescein disodium salt).

The free dye content was measured by CE in 30 mM NaH_2PO_4 buffer of pH set to 6.1. The samples were run in uncoated fused silica capillaries of 25 cm effective length at 20 kV applied voltage and introduced hydrodynamically at 200 mbar·s. On each day, before a set of measurements the capillary was washed with water for one minute, followed by 1 M NaOH for ten minutes, 0.1 M NaOH for three minutes and again water for one minute. Between the runs, the capillary was flushed with 0.1 M NaOH–water–0.1 M NaOH for 1 minute each and with the operating buffer for 2.5 min. A series of calibration solutions corresponding to 5-0.2% of free dye in the product were applied for quantification.

The UV-Vis spectra were recorded in 0.04 M citrate–0.04 M borate–0.04 M phosphate universal buffer and the desired pH set with NaOH solution. The concentration of the conjugate was 0.005 (m/m)%, while the concentration of the free dye was 0.0005 (m/m)%.

Synthesis of xanthene-appended cyclodextrins

(6-Spirolactam rhodamine B-6-deoxy)-β-cyclodextrin (Rho-β-CD): Rhodamine B (160 mg, 0.3 mmol) was dissolved in H_2O (6 mL) and NMM (132 µL, 1.2 mmol), 6-monodeoxy-6-monoamino-β-CD hydrochloride (350 mg, 0.3 mmol), DMT-MM (83 mg, 0.3 mmol) were added in sequence to the pink solution. The mixture was stirred at r.t. for 3 h, concentrated under reduced pressure to half of the volume and precipitated with acetone (100 mL). The precipitate was filtered and washed with acetone (3 × 5 mL) in order to remove the unreacted dye. The crude (360 mg) was purified by chromatography (gradient eluents: CH_3CN/H_2O = 8:2 and CH_3CN/H_2O = 7:3, 6 g of silica gel per 50 mg of crude), the fractions were concentrated under reduced pressure and addition of acetone (50 mL) yielded a pink

precipitate. The solid was filtered, washed with acetone (3 × 2 mL) and drying at 60 °C under reduced pressure (10 mbar) overnight in the presence of P_2O_5 and KOH yielded Rho-β-CD as a slight pink powder (350 mg, 72%).

ESI-MS *m/z* found 1559.3960 $[M+H]^+$, calcd for $C_{70}H_{100}O_{36}N_3$ 1559.6154; *m/z* found 1581.3973 $[M+Na]^+$ calcd for $[C_{70}H_{100}O_{36}N_3$+ Na] 1582.5307.

IR (KBr) ν/cm-1: 3398 (O-H), 2970 (C-H), 1755 (γ-lactam ring, C=O stretching), 1616, 1519, 1429, 1334, 1221, 1122, 1027, 760, 701.

^{1}H-NMR (600 MHz, D_2O): δ 7.90-7.88 (d, 1H), 7.48-7.45 (t, 1H), 7.42-7.40 (t, 1H), 6.72-6.71 (d, 1H), 6.25-6,24 (d, 1H), 6.22 (s, 1H), 6.19-6.17 (d, 1H), 6.00 (s, 1H), 5.64-5.63 (d, 1H), 5.61-5.60 (d, 1H) (aromatic region, see assignments in ESI-1 and ESI-3), 5.05-5.04 (d, 1H), 4.99 (d, 1H), 4.92 (d, 1H), 4.91-4.90 (d, 1H), 4.88-4.87 (d, 2H), 4.78-4.77 (d, 1H), 4.31-2.71 (m, 50H) (partial assignments as shown in Figures 4,5), 1.16-1.14 (t, 6H), 0.82-0.80 (t, 6H) (see assignments in Figures 4,5).

^{13}C-NMR (600 MHz, D_2O): δ 172.62, 156.22, 155.54, 154.97, 152.42, 151.11, 136.08, 132.80, 132.12, 131.80, 130.60, 125.69, 125.47, 112.60, 111.27, 107.21, 105.68, 105.29, 105.20, 104.88, 104.61, 104.46, 103.80, 101.56, 100.23, 85.94, 83.40, 83.07, 82.99, 82.29, 80.87, 76.40, 76.33, 75.96, 75.88, 75.71, 75.54, 75.06, 74.93, 74.70, 74.66, 74.60, 74.29, 74.15, 74.09, 74.05, 73.90, 73.79, 62.91, 62.78, 62.19, 61.91, 61.29, 61.09, 47.33, 46.87, 43.76, 15.02, 14.20 (partial assignments as shown in Figure 5 and ESI-3).

Free rhodamine content based on TLC: <0.1 % (w/v).

Free rhodamine content based on CE: <0.1 % (w/v).

(6-Fluoresceinyl-carboxamido-6-deoxy)-β-Cyclodextrin (Flu-β-CD): Fluorescein disodium salt (110 mg, 0.3 mmol) was dissolved in H_2O (6 mL) and NMM (132 μL, 1.2 mmol), 6-monodeoxy-6-monoamino-β-CD hydrochloride (350 mg, 0.3 mmol), DMT-MM (83 mg, 0.3

mmol) were added in sequence to the yellow solution. The mixture was stirred at r.t. for 3 h, concentrated under reduced pressure to half of the volume and precipitated with acetone (100 mL). The precipitate was filtered and washed with acetone (3 × 5 mL) in order to remove the dye-related by-products. The crude (390 mg) was purified by chromatography (eluent: CH_3CN/H_2O/ammonium hydroxide (25%) = 10:5:1, 10 g of silica gel per 50 mg of crude), the fractions were concentrated under reduced pressure and addition of acetone (50 mL) yielded an orange precipitate. The solid was filtered, washed with acetone (3 × 2 mL) and drying at 60 °C under reduced pressure (10 mbar) overnight in the presence of P_2O_5 and KOH yielded Flu-β-CD as a slight orange powder (154 mg, 35%).

ESI-MS m/z found 1449.1524 $[M+H]^+$, calcd for $C_{62}H_{81}O_{38}N$ 1448.2904; m/z.

^{1}H-NMR (600 MHz, D_2O): δ 7.83-7.82 (d, 1H), 7.47-7.42 (dt, 2H), 6.63.-6.61 (d, 1H), 6.61-6.60 (d, 1H), 6.55-6,53 (dd, 1H), 6.48-6.46 (d, 1H), 6.43 (d, 1H), 6.39-6.37 (dd, 1H), 6.32-6.30 (d, 1H), (aromatic region, see assignments in ESI-16 and ESI-20) 5.05-5.04 (d, 1H), 4.96 (d, 1H) 4.92-4.90 (m, 3H), 4.84-4.83 (d, 1H), 4.81-4.80 (d, 1H), 4.19-2.45 (m, 42H) (partial assignments as shown in Fig. 7, ESI-18, ESI-21, ESI-24).

^{13}C-NMR (600 MHz, D_2O): δ 174.07, 161.09, 160.01, 156.50, 155.05, 154.40, 136.56, 131.99, 131.92, 130.56, 130.36, 125.84, 125.24, 115.70, 114.19, 112.09, 111.49, 106.02, 105.06, 104.98, 104.81, 104.40, 104.30, 103.29, 101.14, 85.50, 83.69, 83.65, 83.52, 83.42, 83.26, 82.58, 81.77, 80.75, 76.37, 75.92, 75.81, 75.64, 75.40, 75.33, 75.24, 75.13, 74.91, 74.77, 74.64, 74.56, 74.37, 74.21, 74.09, 74.04, 73.65, 73.50, 62.93, 62.33, 62.08, 61.49, 61.46, 60.89, 44.16 (partial assignments as shown in ESI-20, ESI-21, ESI-23 and ESI-24).

Free fluorescein content based on TLC: <0.1 % (w/v).

Free fluorescein content based on CE: <0.1 % (w/v).

Spectra of synthesized compounds

Figure S1

Rho-β-CD

^{1}H-NMR Spectrum

(expansion of aromatic region with assignment)

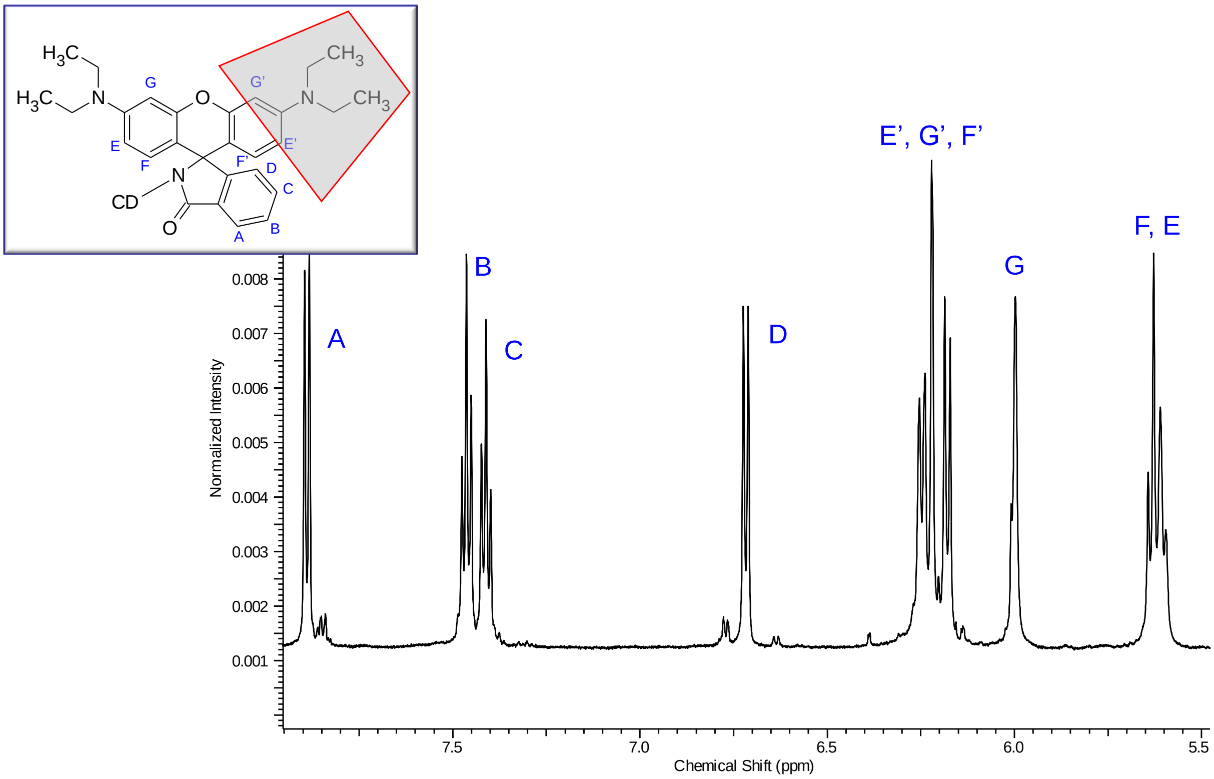

Figure S2

Rho-β-CD

COSY Spectrum

(expansion of aromatic region)

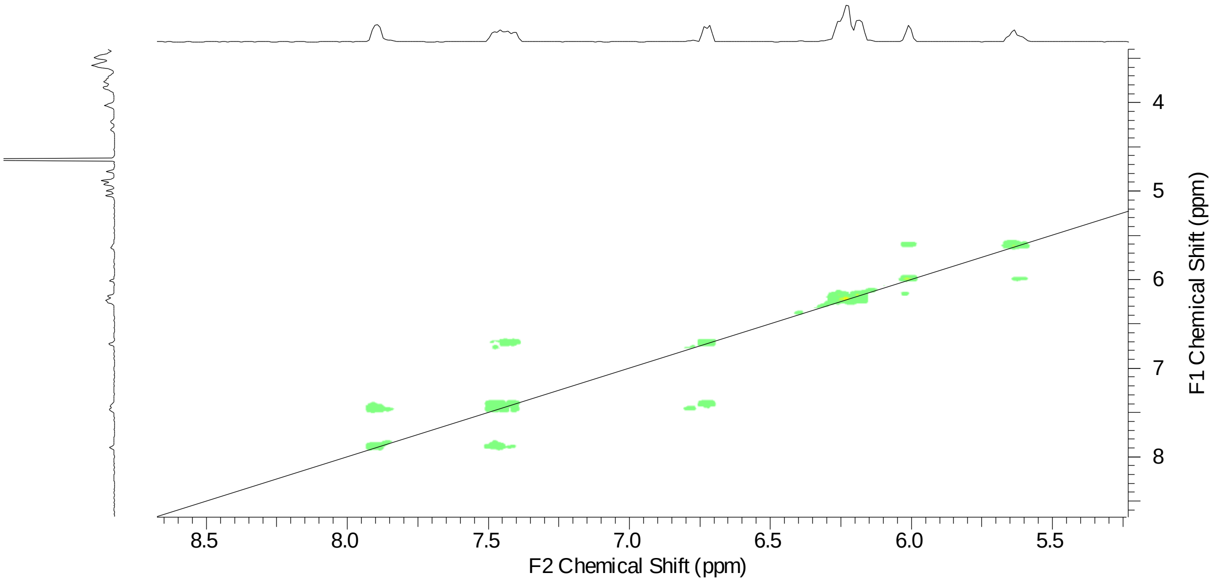

Figure S3

Rho-β-CD

DEPT-ed HSQC Spectrum

(expansion of aromatic region with assignment)

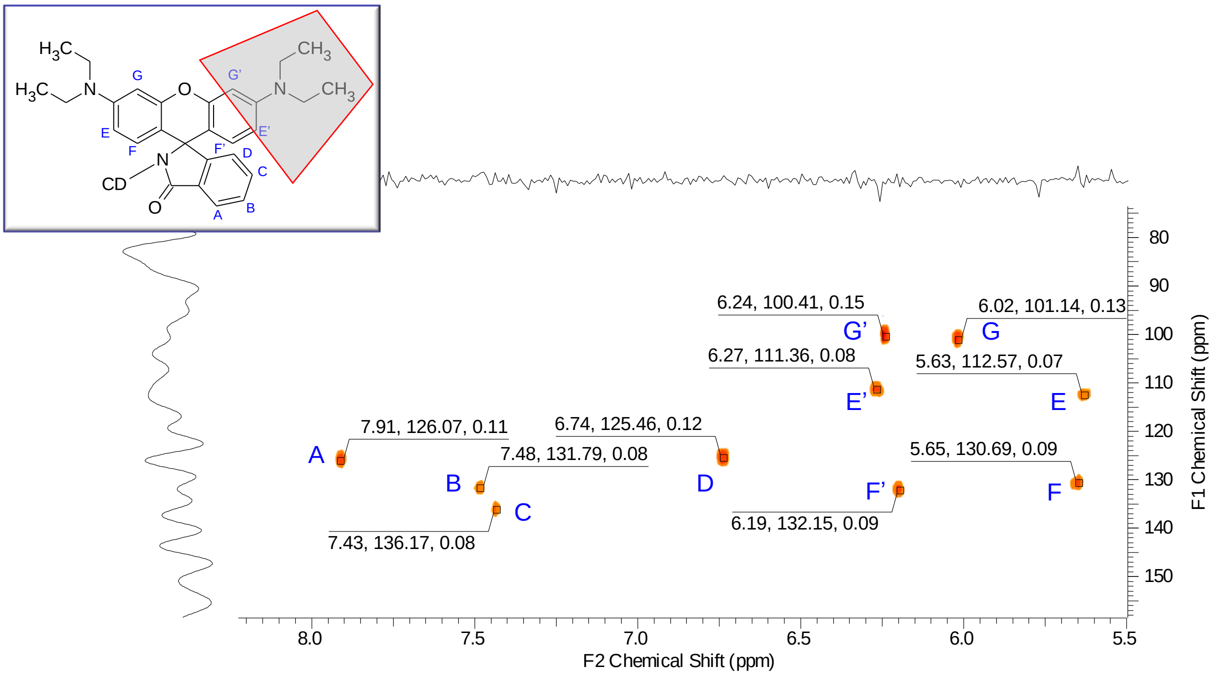

Figure S4

Rho-β-CD

^{1}H-NMR Spectrum

(expansion of anomeric region)

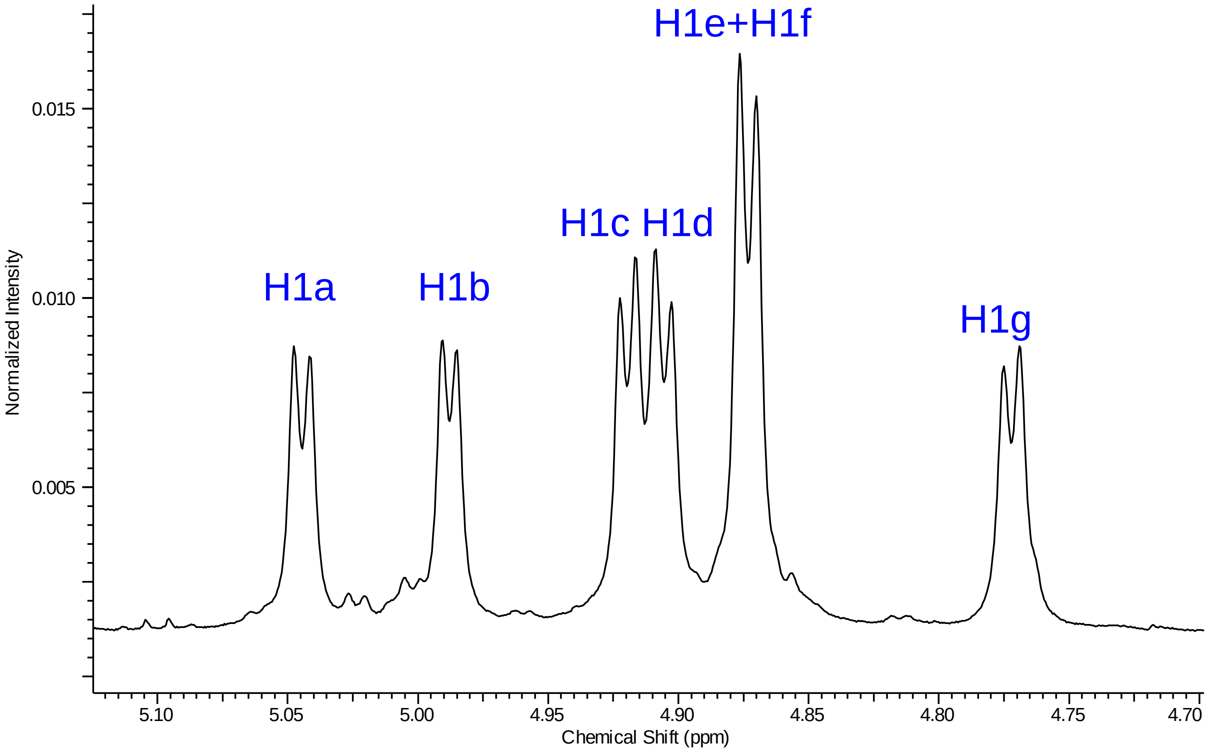

Figure S5

Rho-β-Lactone

^{1}H-NMR Spectrum

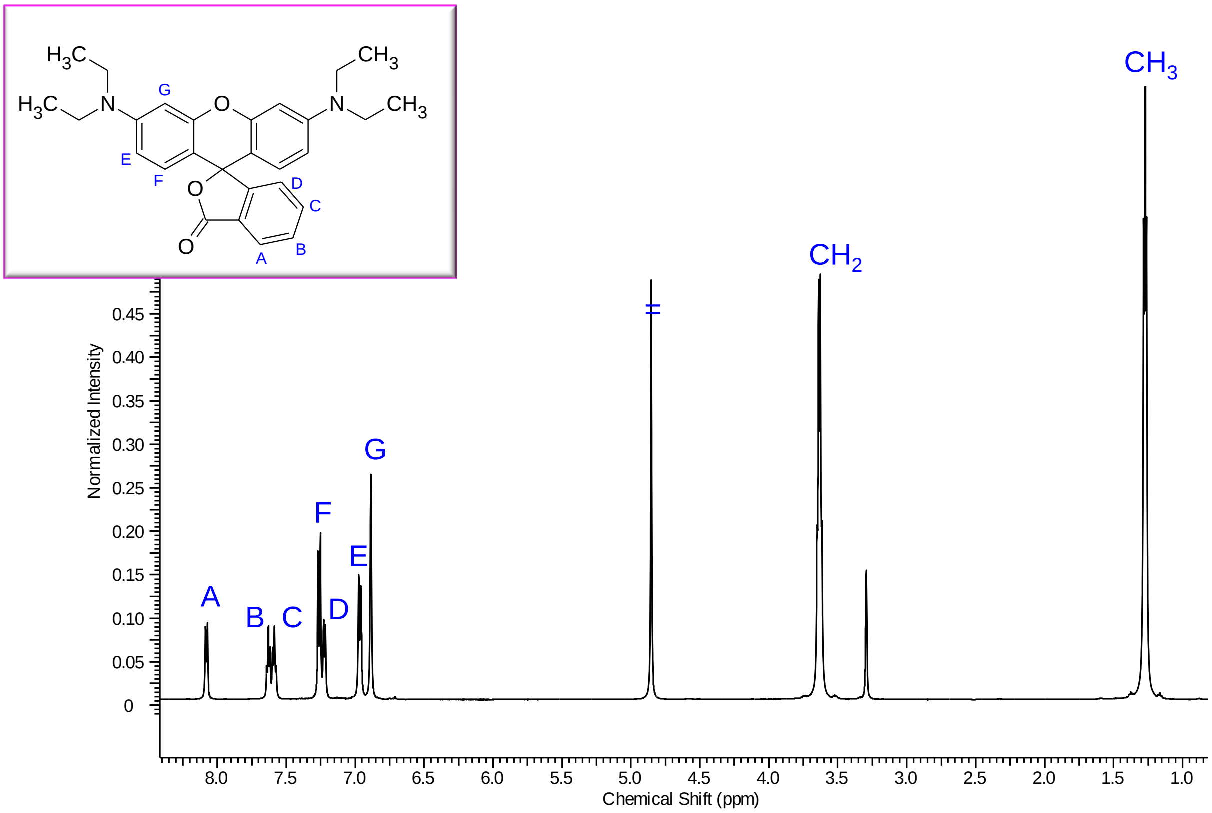

Figure S6

Rho-β HCl

^{1}H-NMR Spectrum

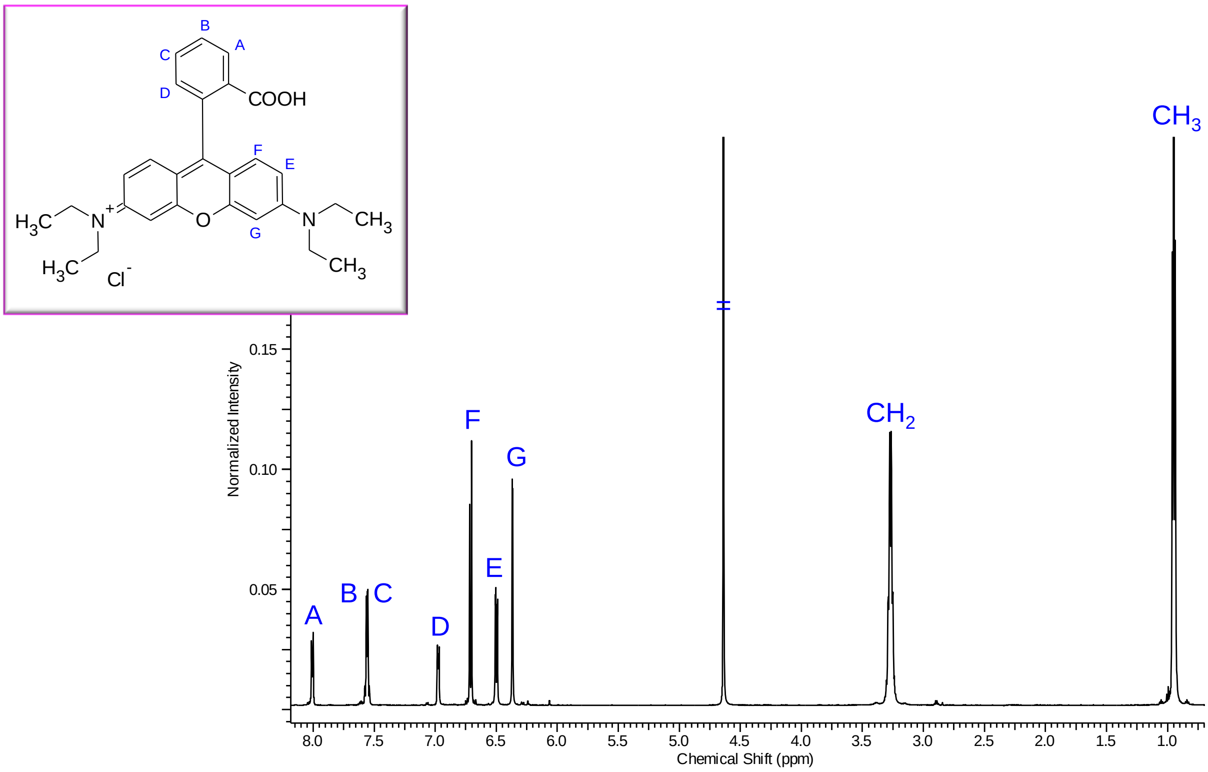

Figure S7

Rho-β-CD

^{1}H-NMR Spectrum in deuterated DMSO

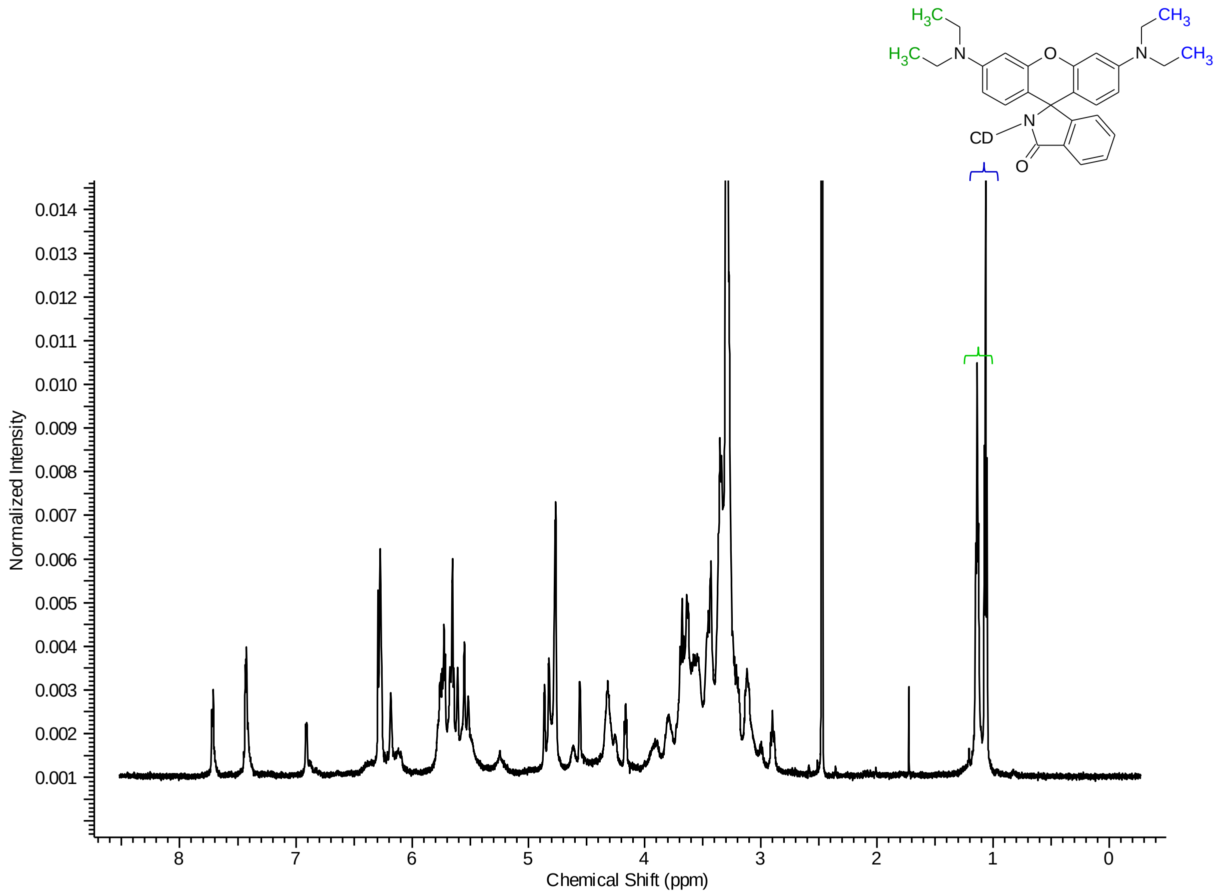

Figure S8

Rho-β-CD

DEPT-ed HSQC Spectrum

(with expansion of anomeric region)

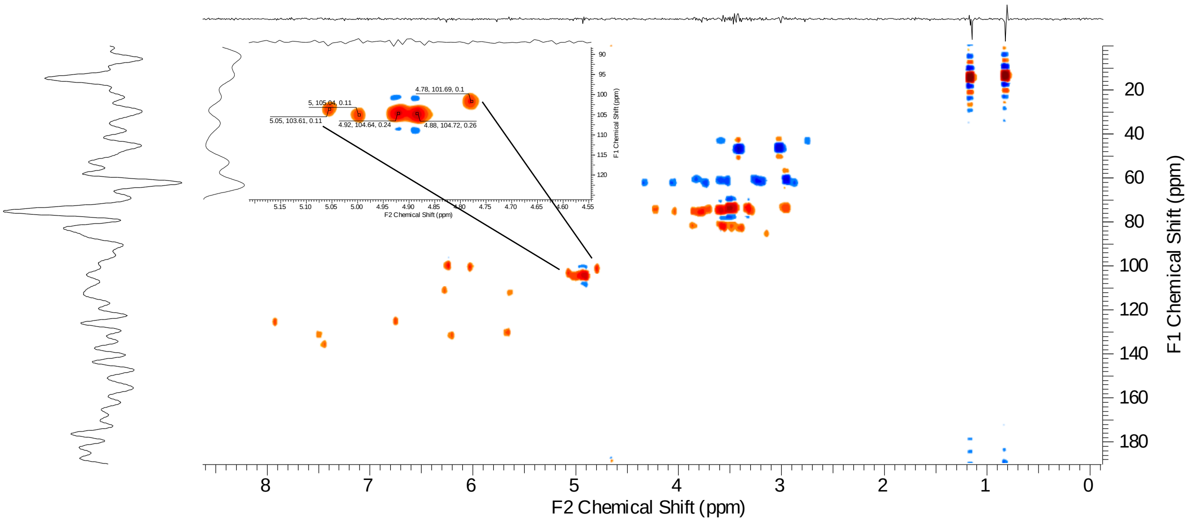

Figure S9

Rho-β-CD

2D TOCSY Spectrum

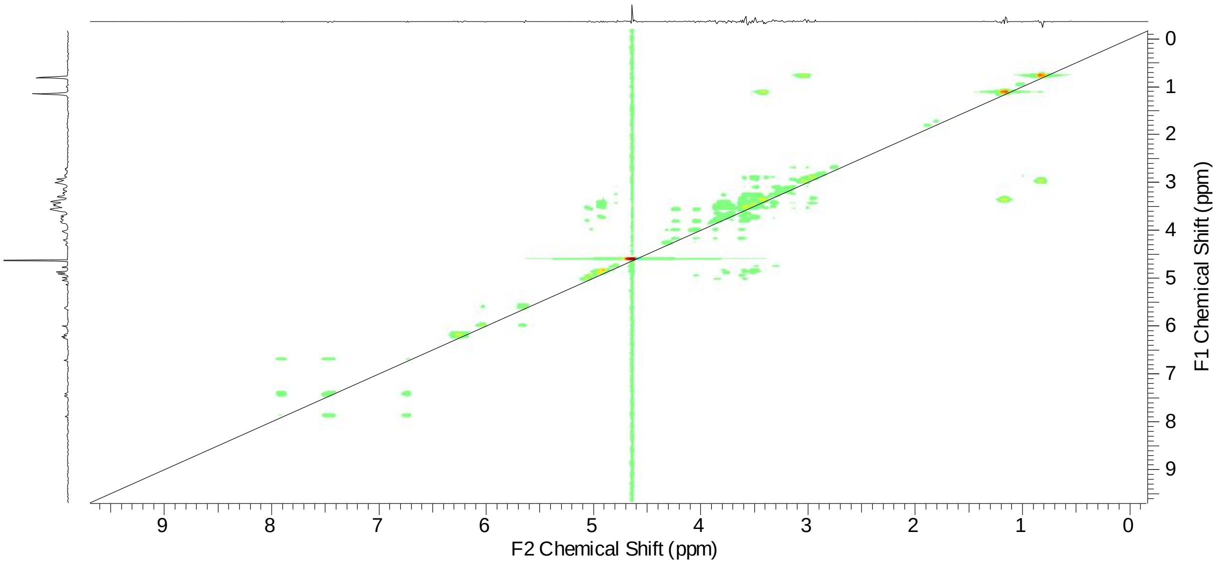

Figure S10

Rho-β-CD

^{13}C Spectrum

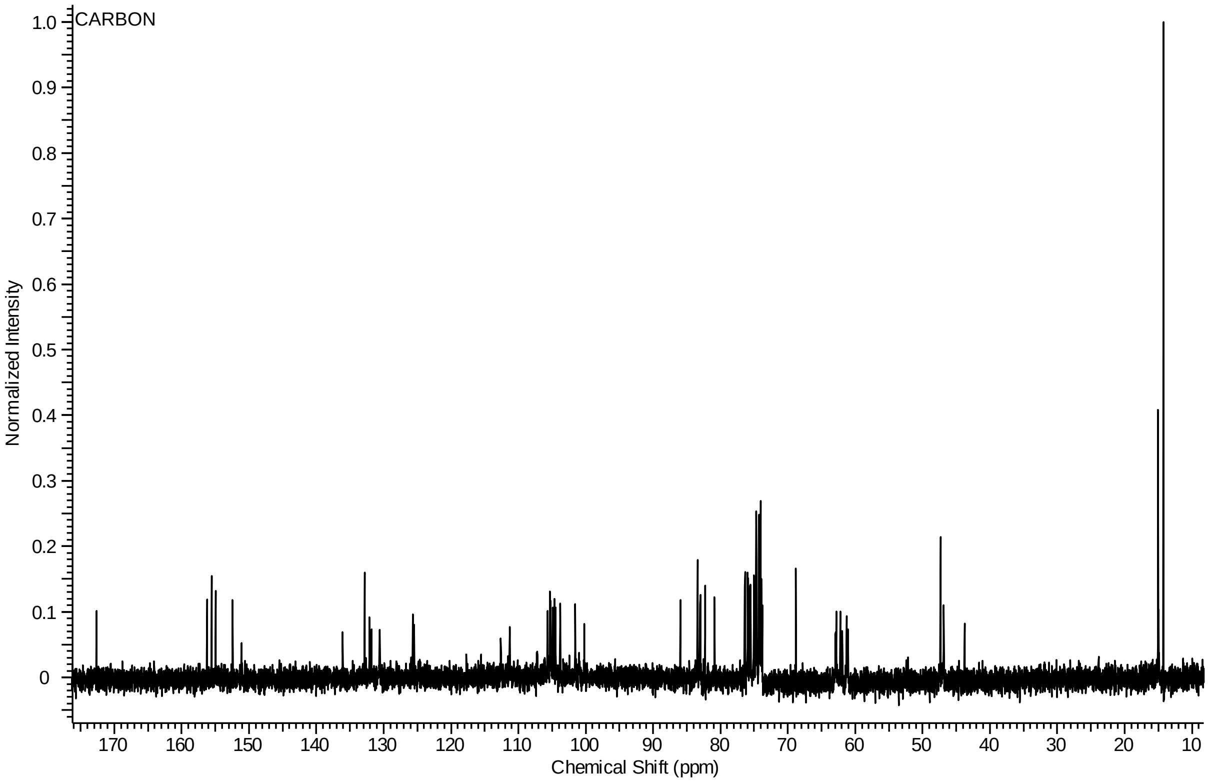

Figure S11

Rho-β-CD

2D ROESY Spectrum

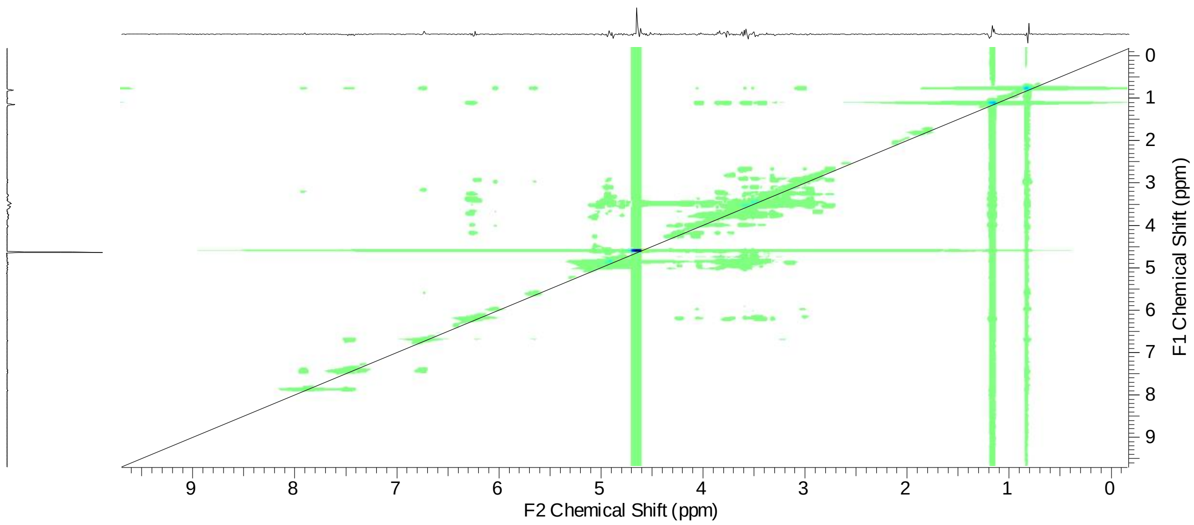

Figure S12

Rho-β-CD

2D ROESY Spectrum

(expansion with peak picking)

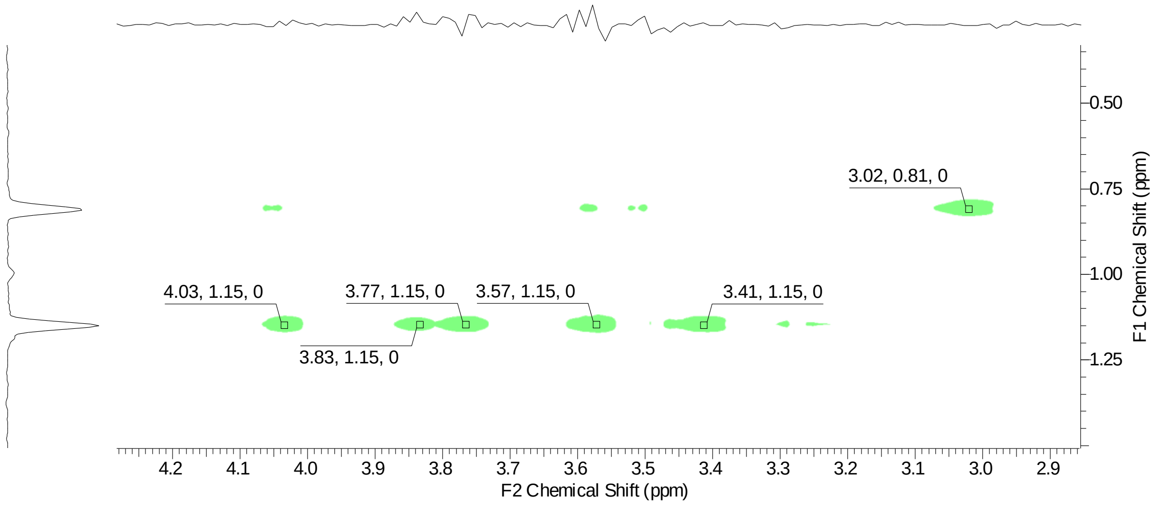

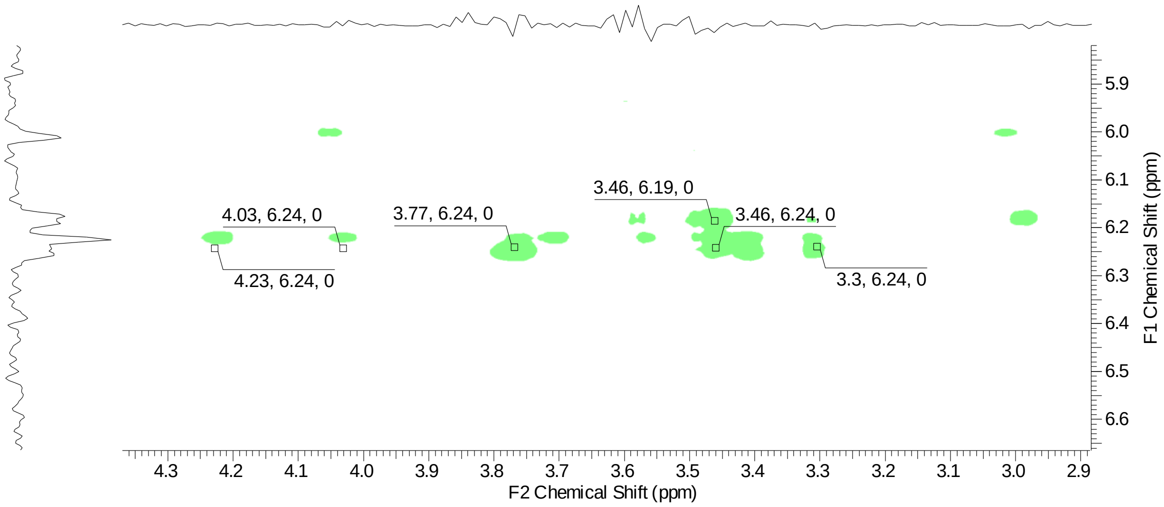

Figure S13

Rho-β-CD

2D ROESY Spectrum

(expansion with peak picking)

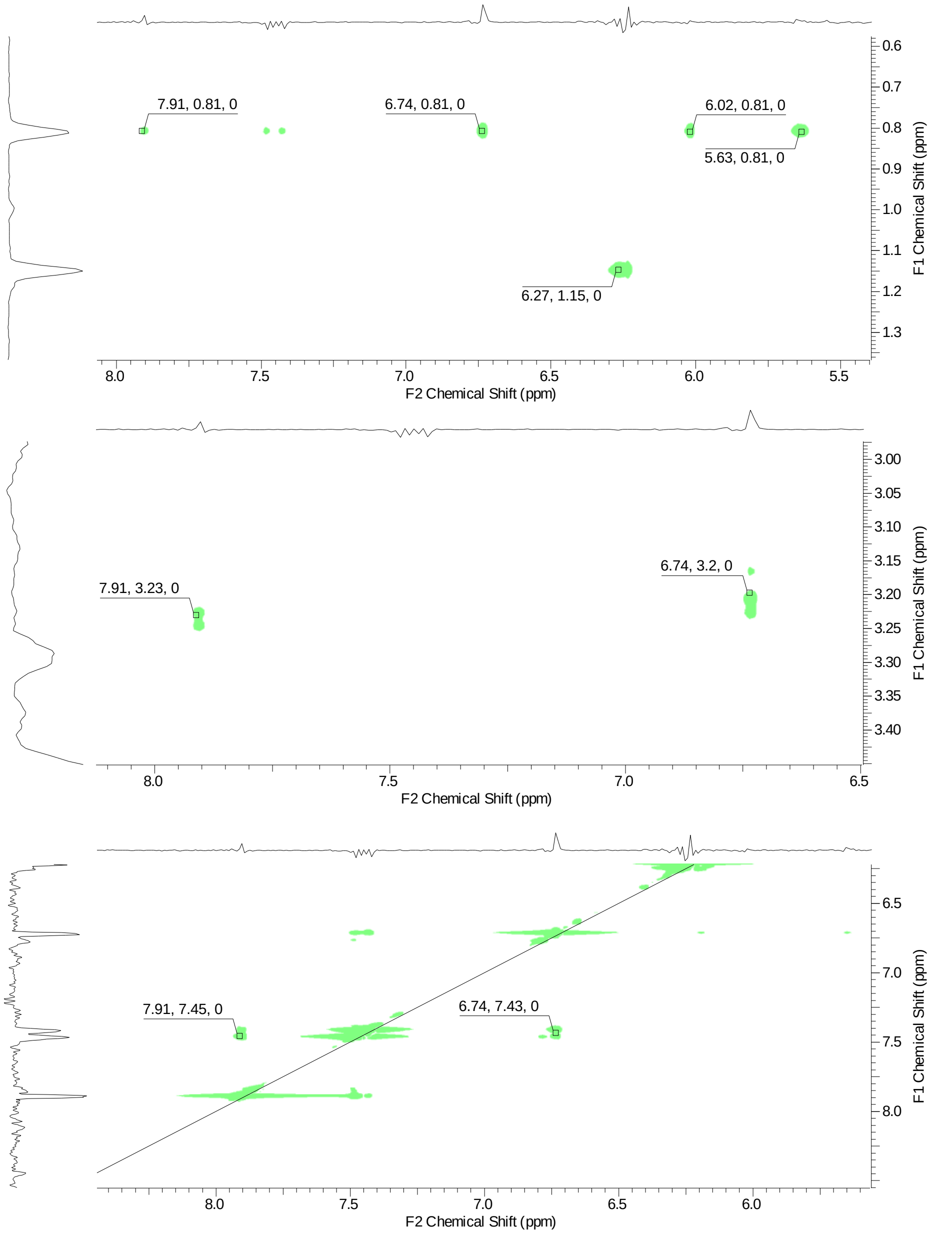

Figure S14

Rho-β-CD

DEPT-ed HSQC Spectrum

(expansion with peak picking)

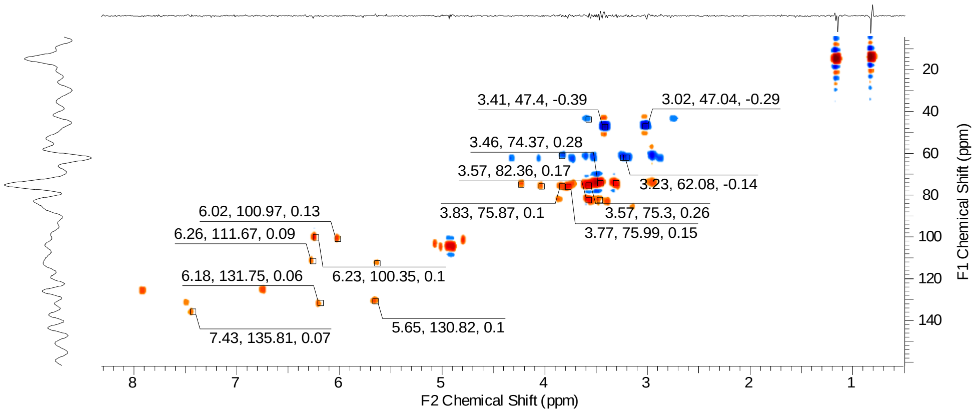

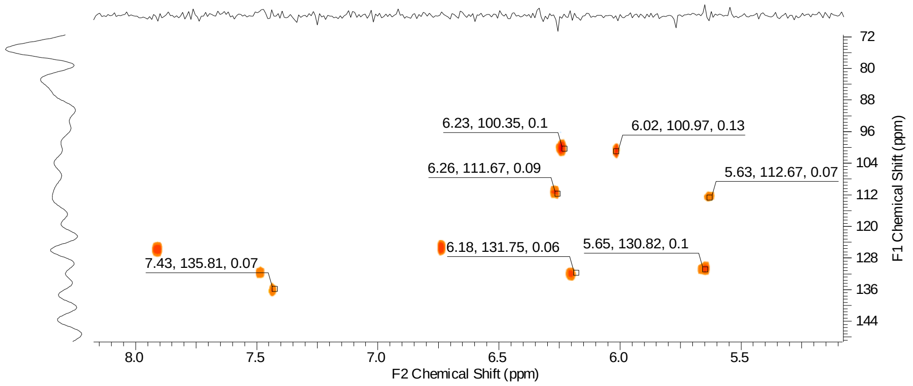

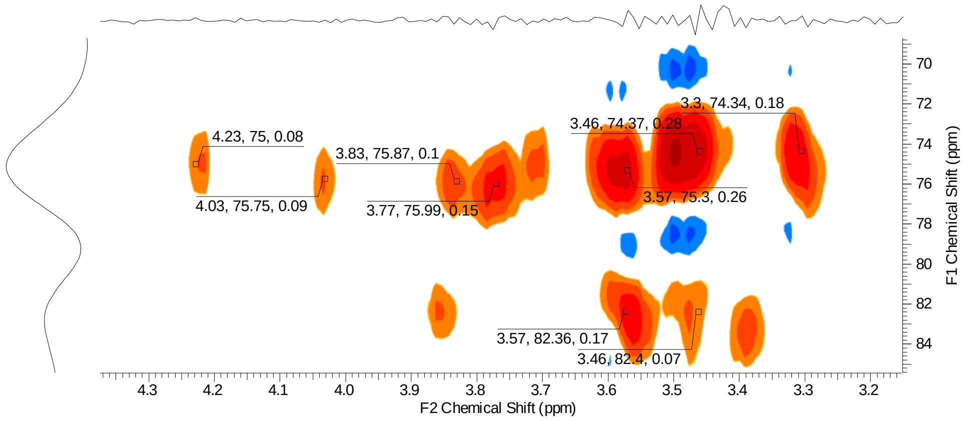

Figure S15

Rho-β-CD

IR Spectra

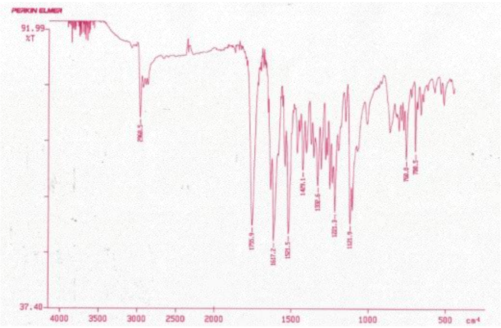

Rho B Lactone

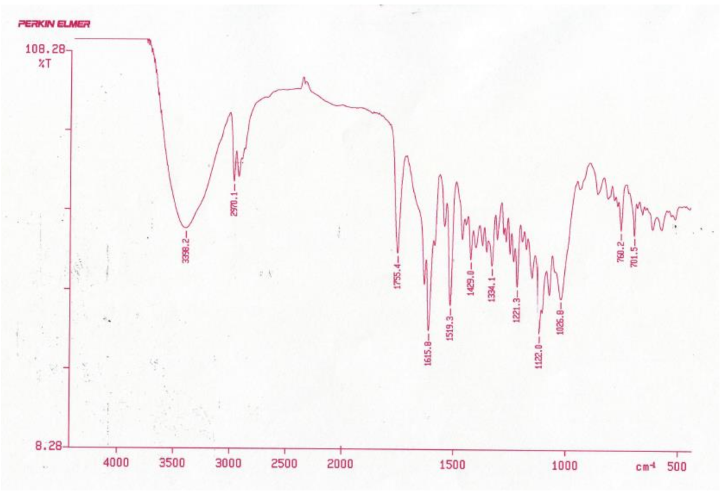

Rho-βCD

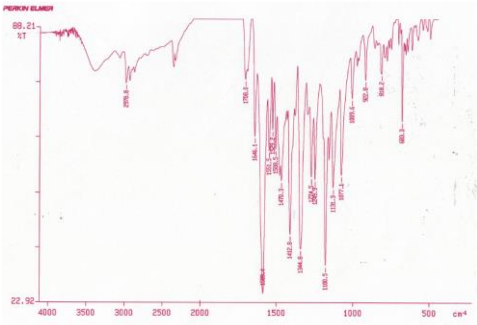

Rho B·HCl

Figure S16

Rho-β-CD

Uv-Vis Spectra

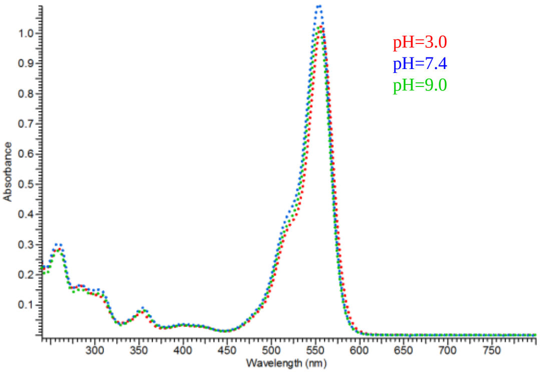

Rhodamine B·HCl

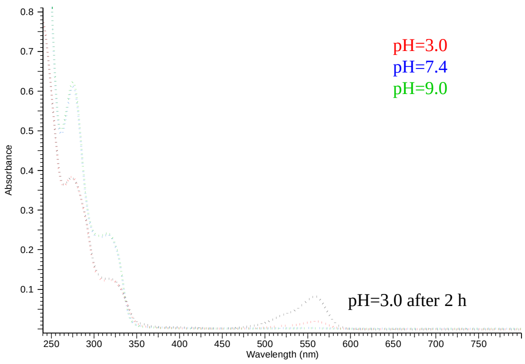

Rho-βCD

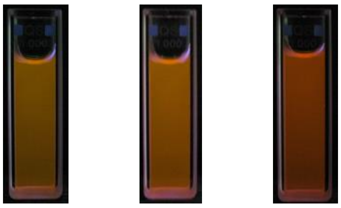

Rhodamine B solution under irradiation at 366 nm
pH=9.0 pH=7.4 pH=3.0

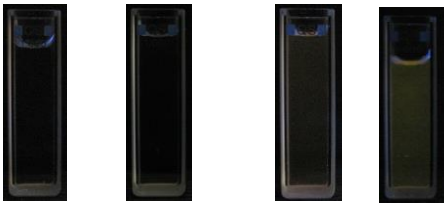

Rho-βCD solution under irradiation at 366 nm
pH=9.0 pH=7.4 pH=3.0 pH=3.0 after 2 h

Figure S17

Flu-β-CD

TLCs and proposed reaction scheme for the fluorescein related by-products formation

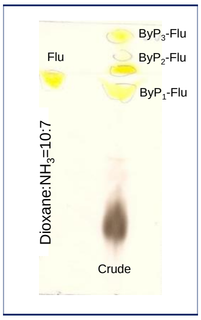

Separation of fluorescein related byproducts

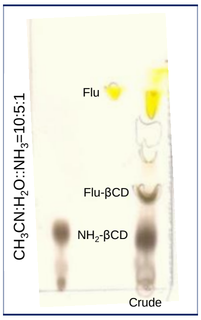

Separation of product from starting material

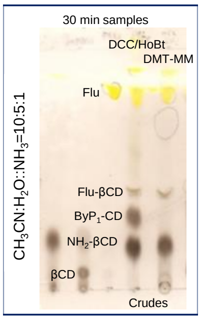

Comparison between DCC/HOBt organic conditions and DMT-MM/NMM aqueous conditions

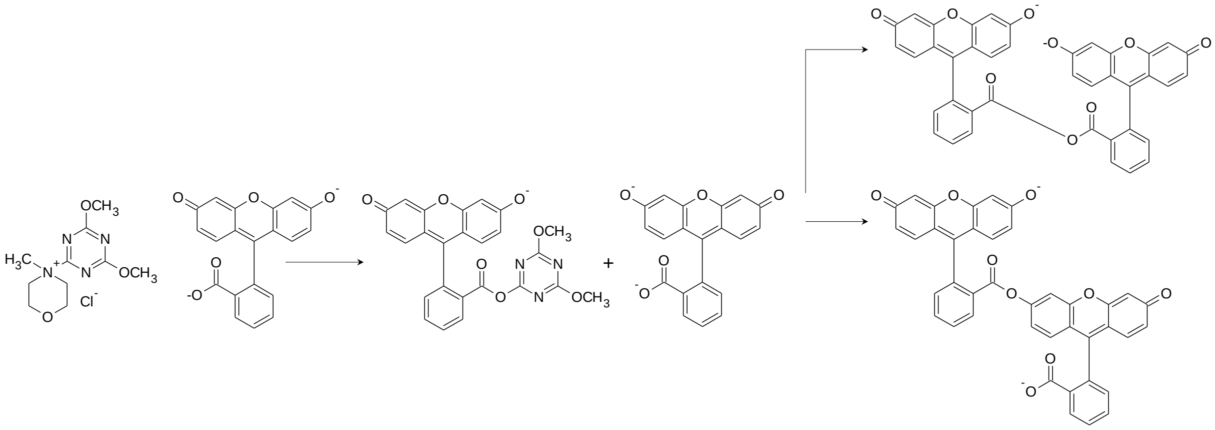

Figure S18

Flu-β-CD

Uv-Vis Spectra

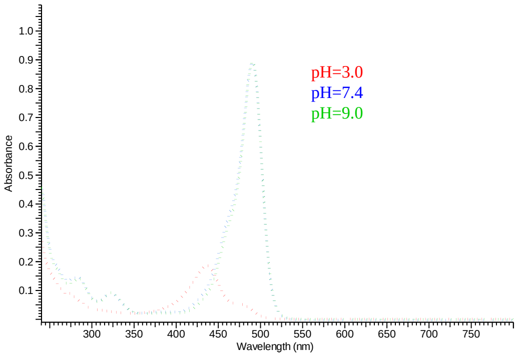

Fluorescein sodium salt

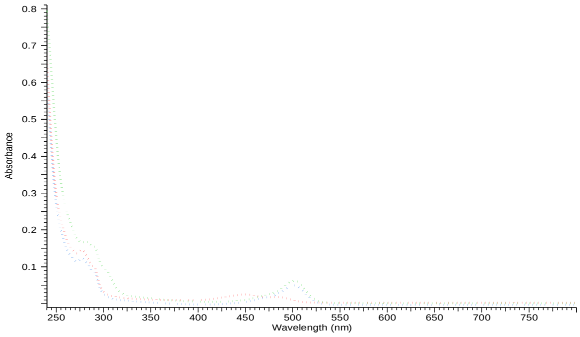

Flu-βCD

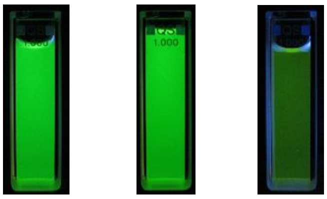

Fluorescein solution under irradiation at 366 nm
pH=9.0 pH=7.4 pH=3.0

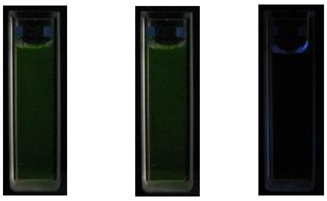

Flu-βCD solution under irradiation at 366 nm
pH=9.0 pH=7.4 pH=3.0

Figure S19

Flu-β-CD

^{1}H-NMR Spectrum

(expansions of aromatic region with assignment)

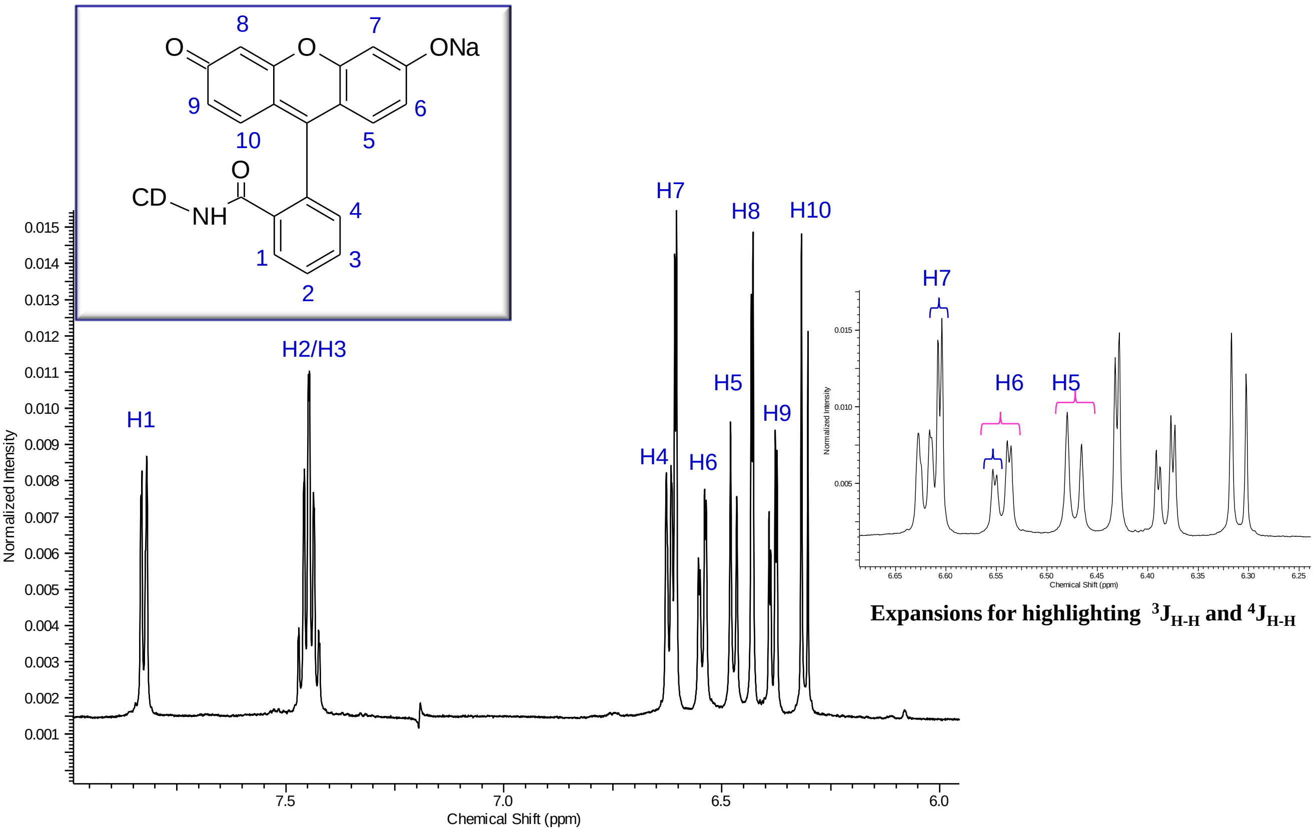

Figure S20

Fluorescein sodium salt

^{1}H-NMR and DEPT-ed HSQC Spectra

(with assignment)

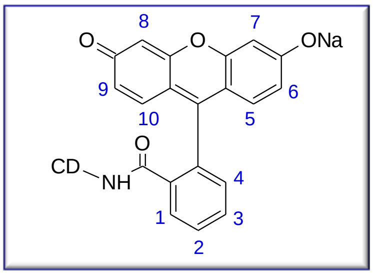

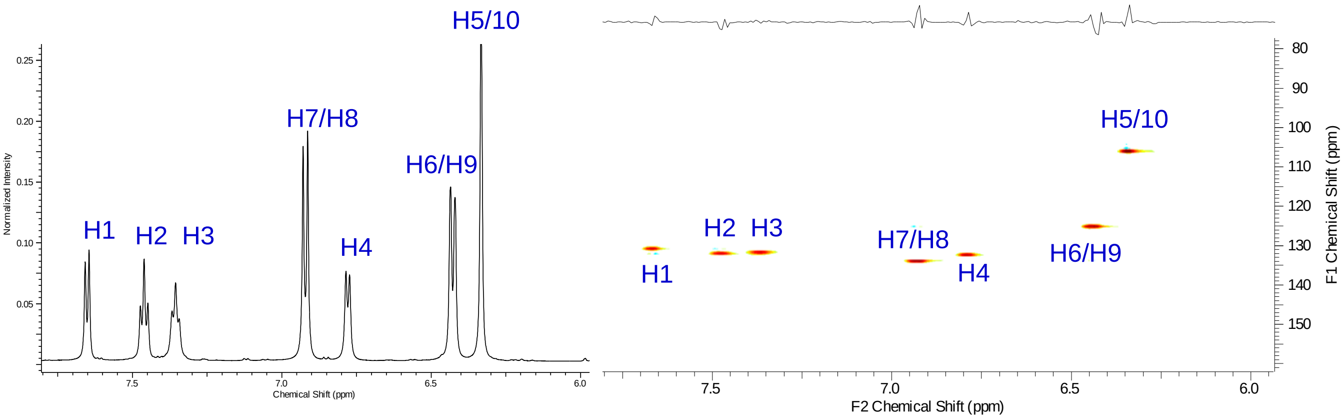

Figure S21

Flu-β-CD

COSY Spectrum

(expansions of aromatic and anomeric region with peak picking)

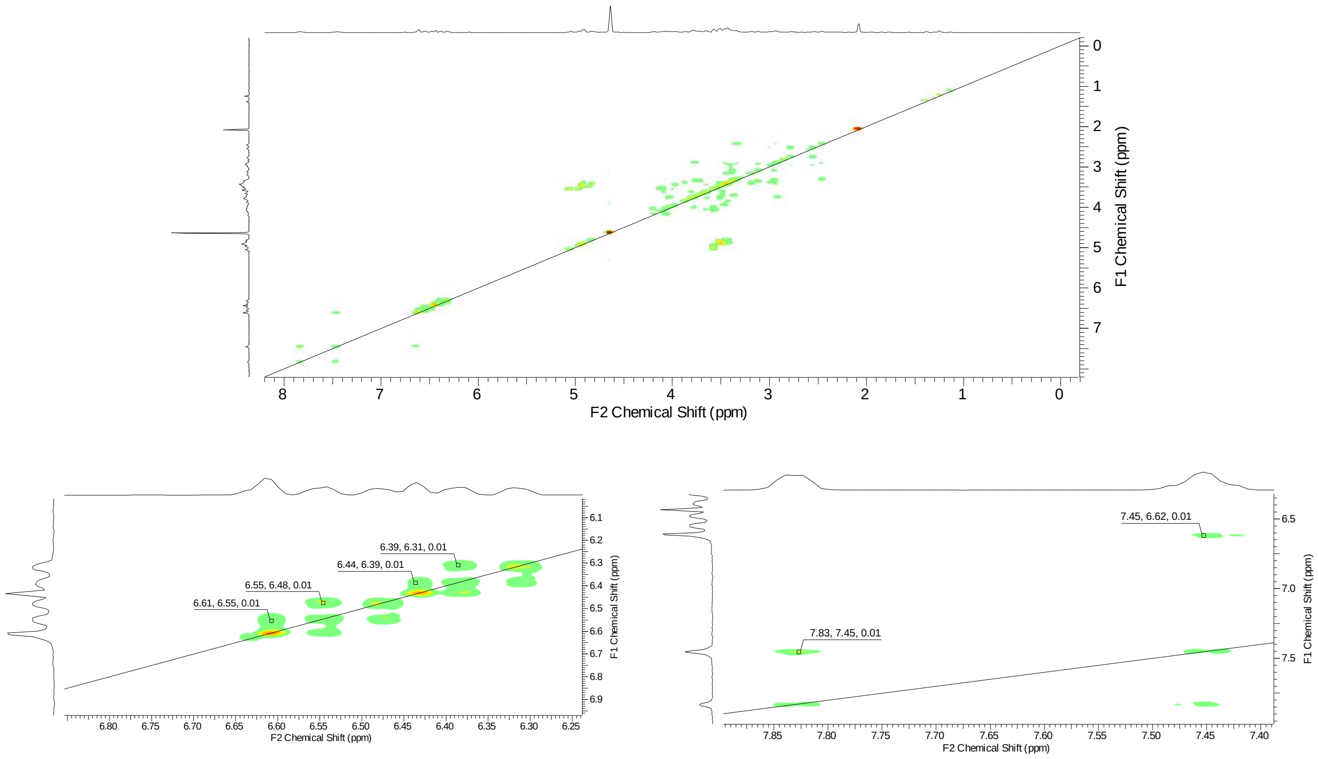

Figure S22

Flu-β-CD

COSY Spectrum

(expansions of aromatic and anomeric region with peak picking

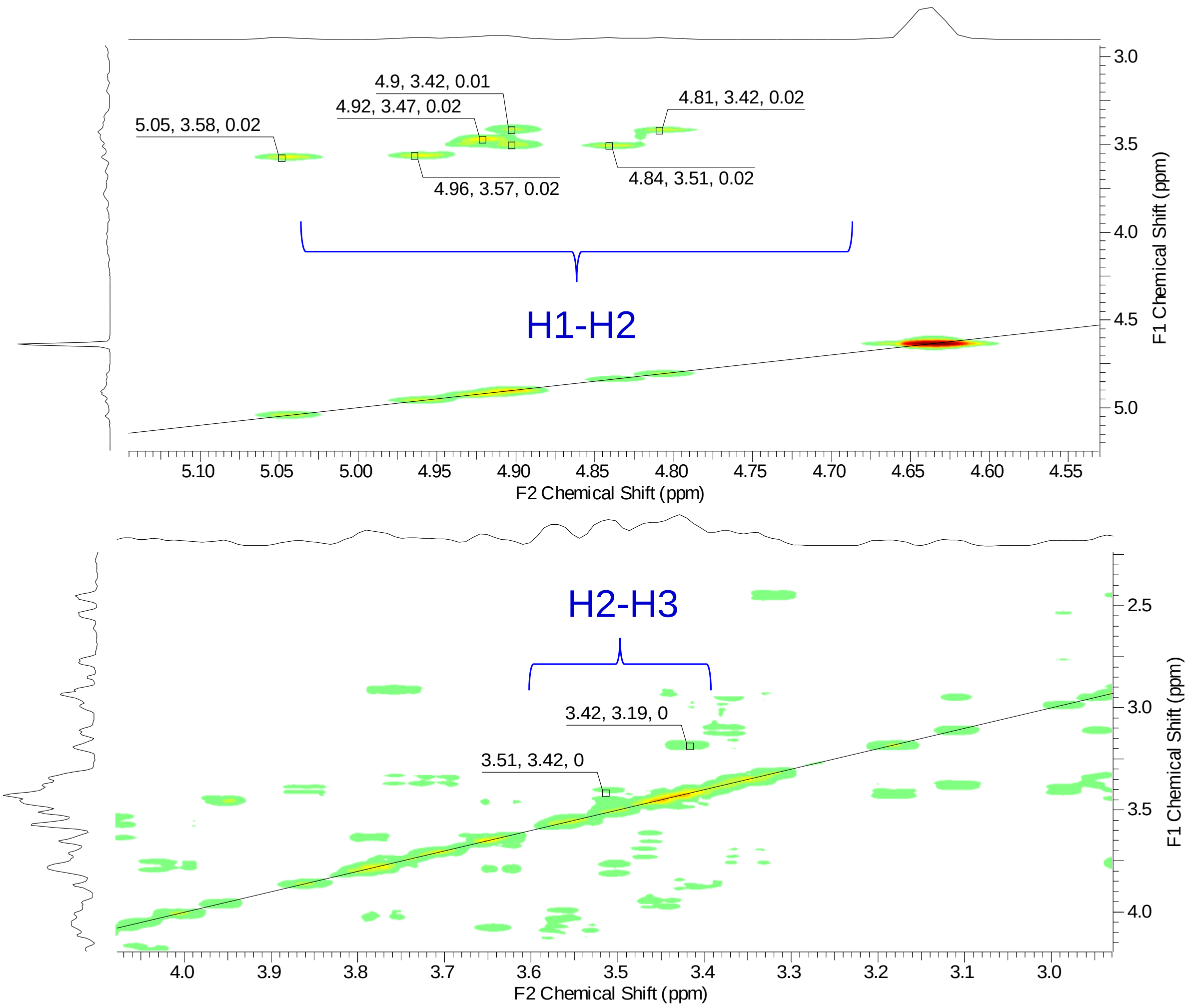

Figure S23

Flu-β-CD

DEPT-ed HSQC Spectrum

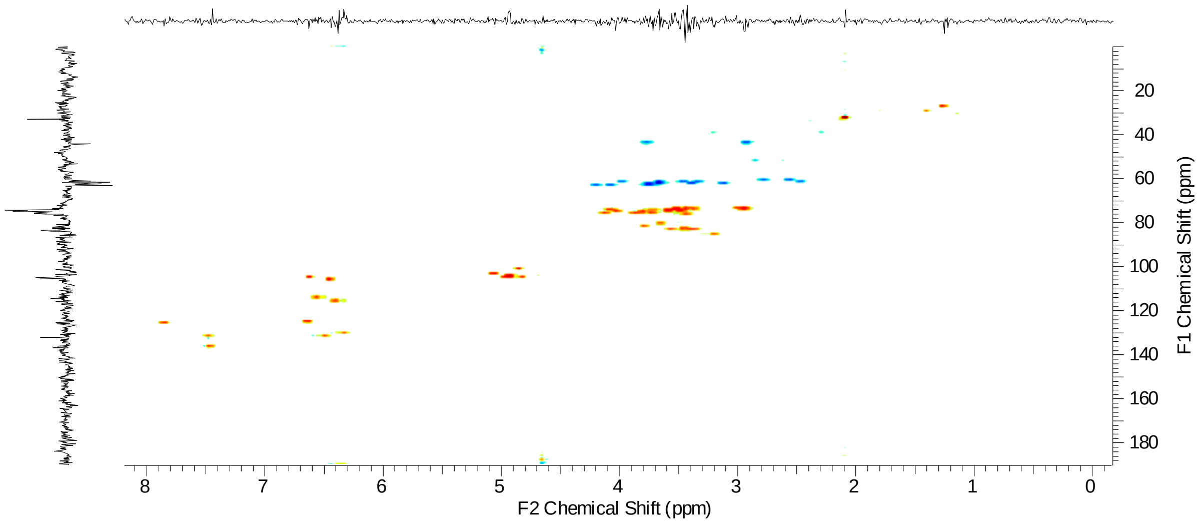

Figure S24

Flu-β-CD

DEPT-ed HSQC Spectrum

(expansion of aromatic region with peak picking and full assignment)

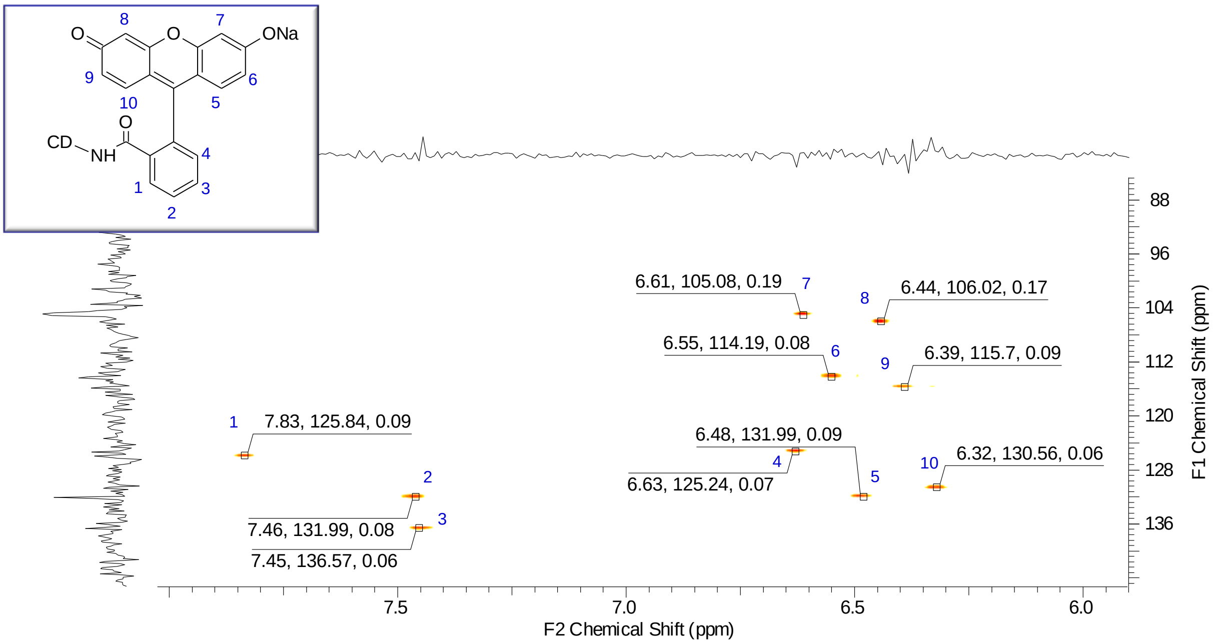

Figure S25

Flu-β-CD

DEPT-ed HSQC Spectrum

(expansion of core region with peak picking and partial assignment)

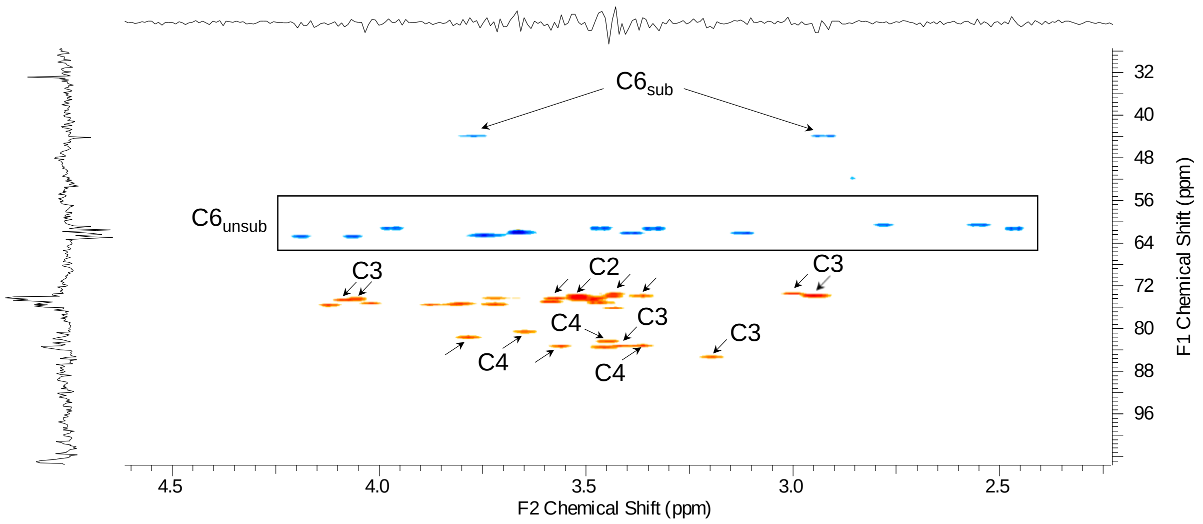

(Assignment based on a combination of COSY, TOCSY, HMBC)

Figure S26

Flu-β-CD

2D TOCSY Spectrum

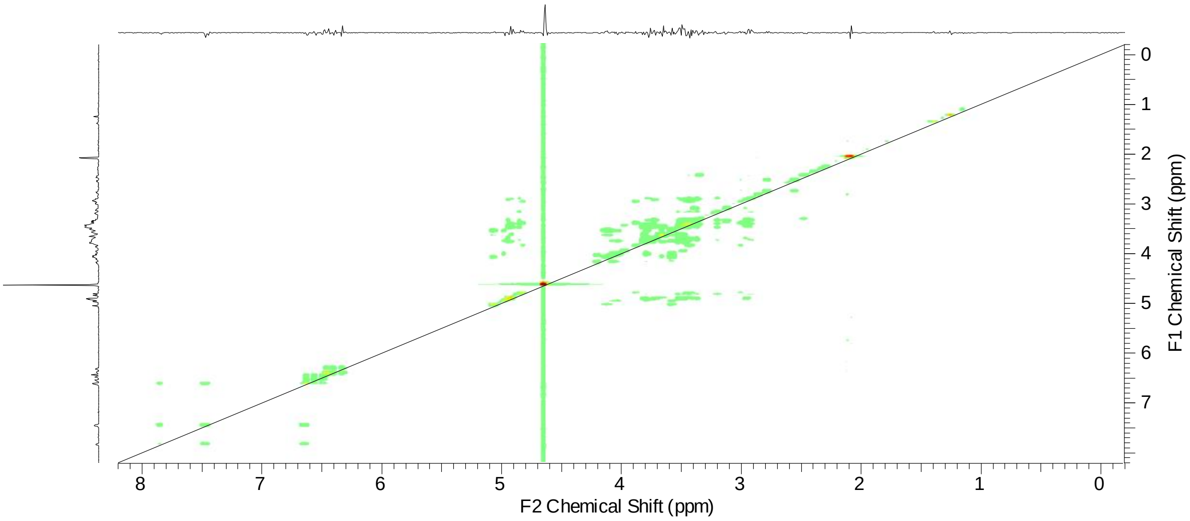

Figure S27

Flu-β-CD

HMBC Spectrum

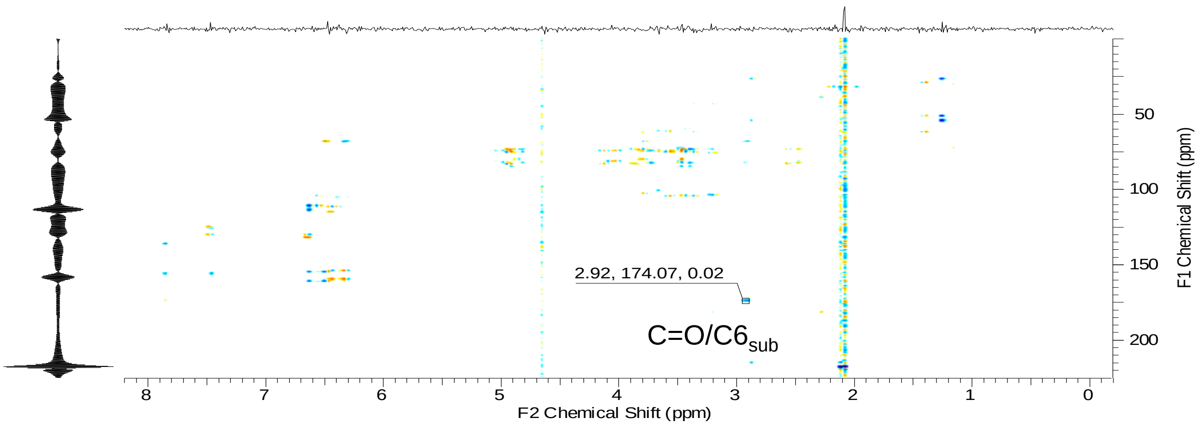

Figure S28

Flu-β-CD

HMBC Spectrum

(selected band 60-86 ppm)

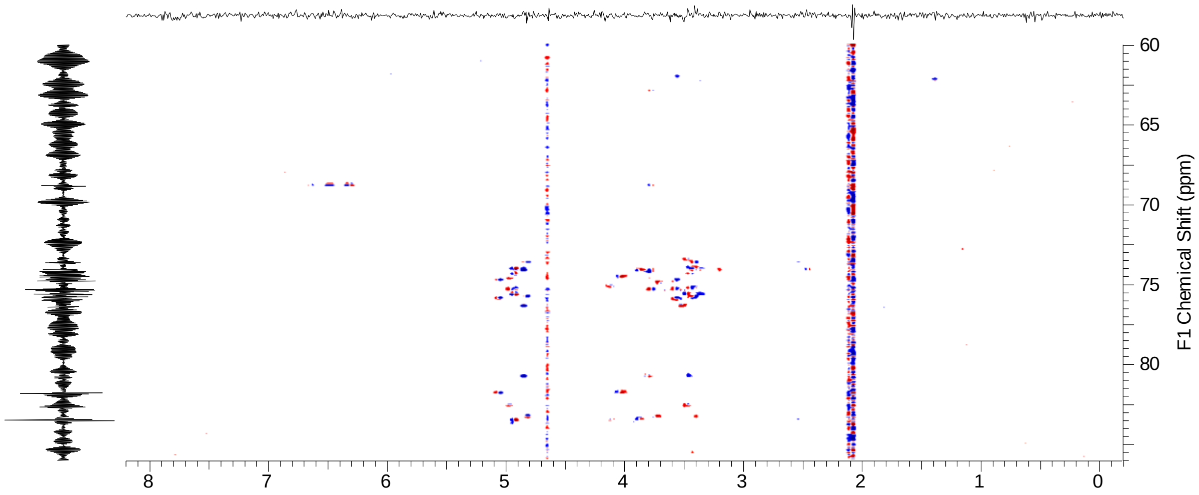

Figure S29

Flu-β-CD

HMBC Spectrum

(expansion on H1-C4 cross peaks of selected band 60-86 ppm)

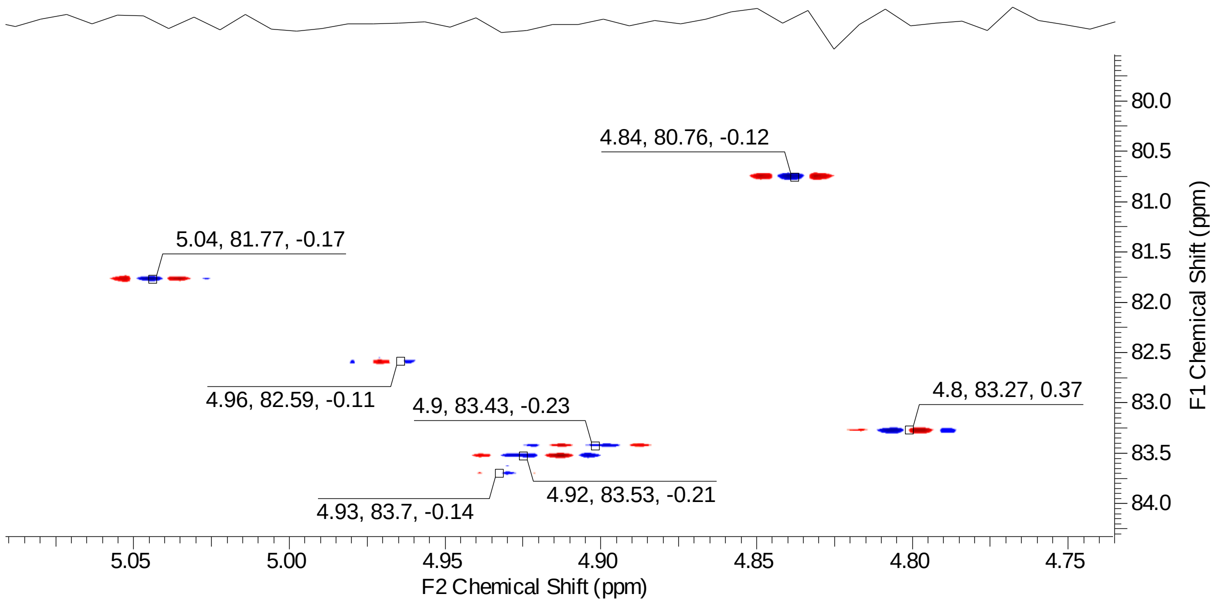

Figure S30

Flu-β-CD

^{13}C Spectrum

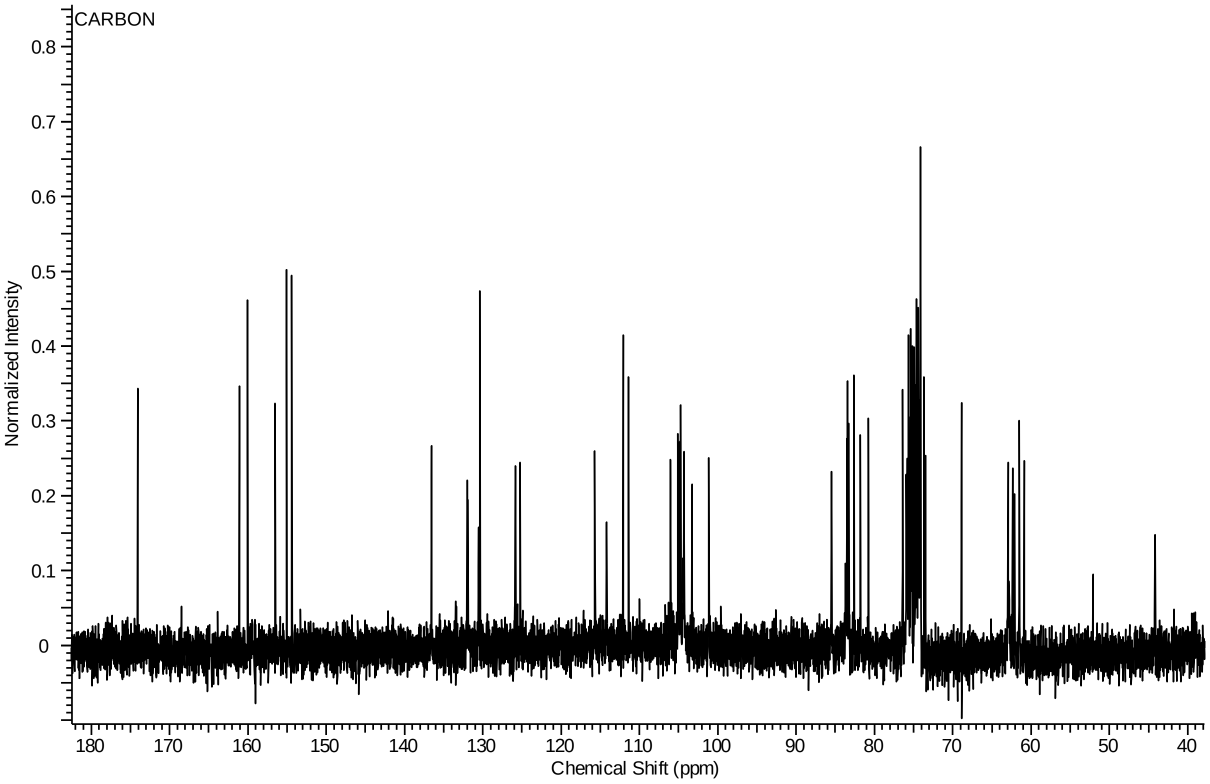

Figure S31

Flu-β-CD

2D ROESY Spectrum

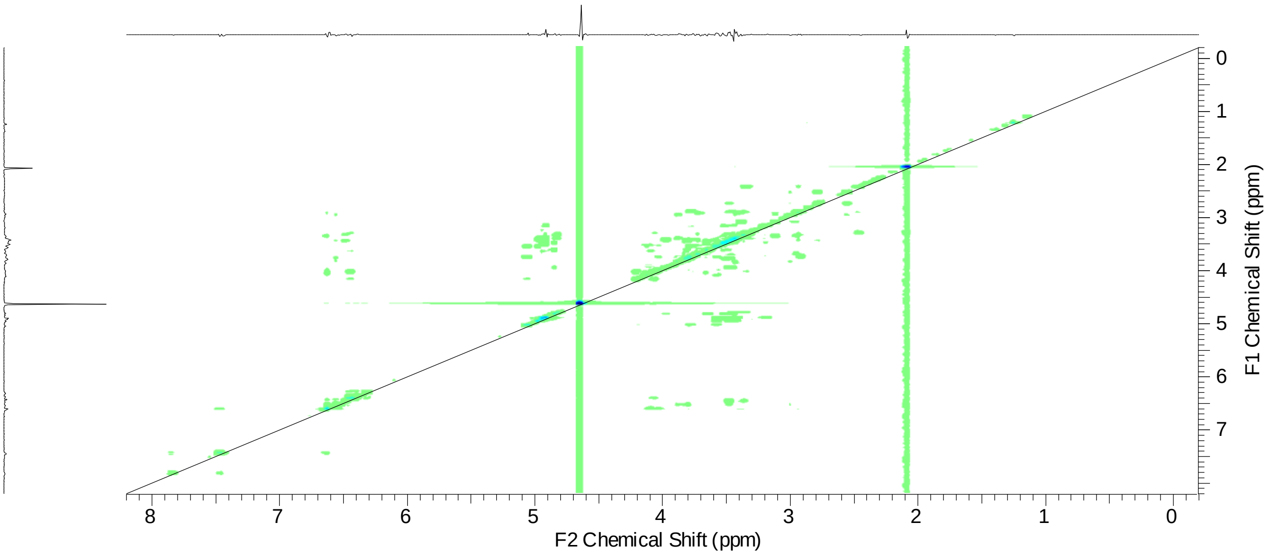

Figure S32

Flu-β-CD

2D ROESY Spectrum

(expansion with peak picking)

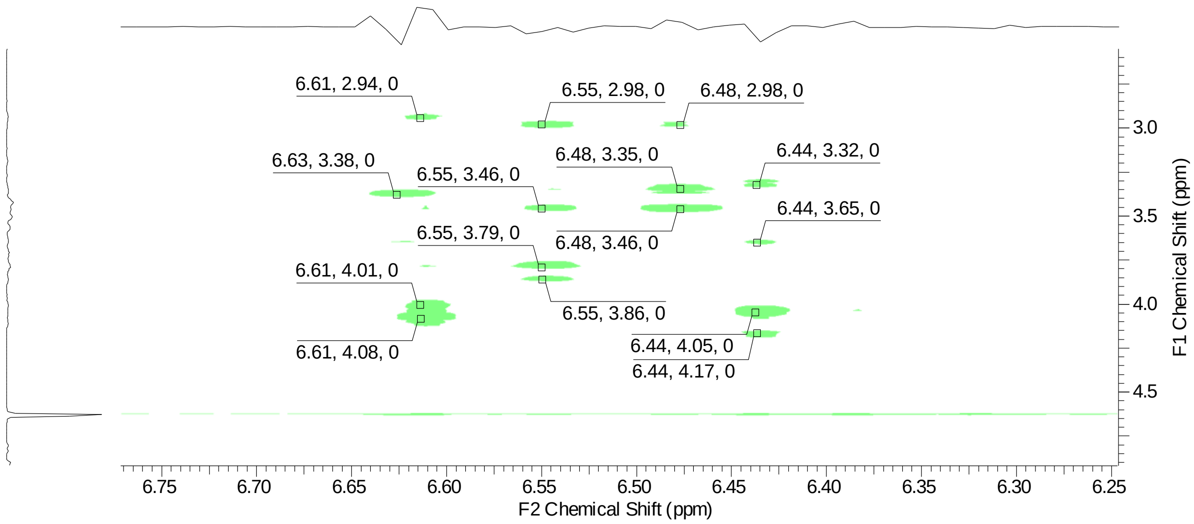

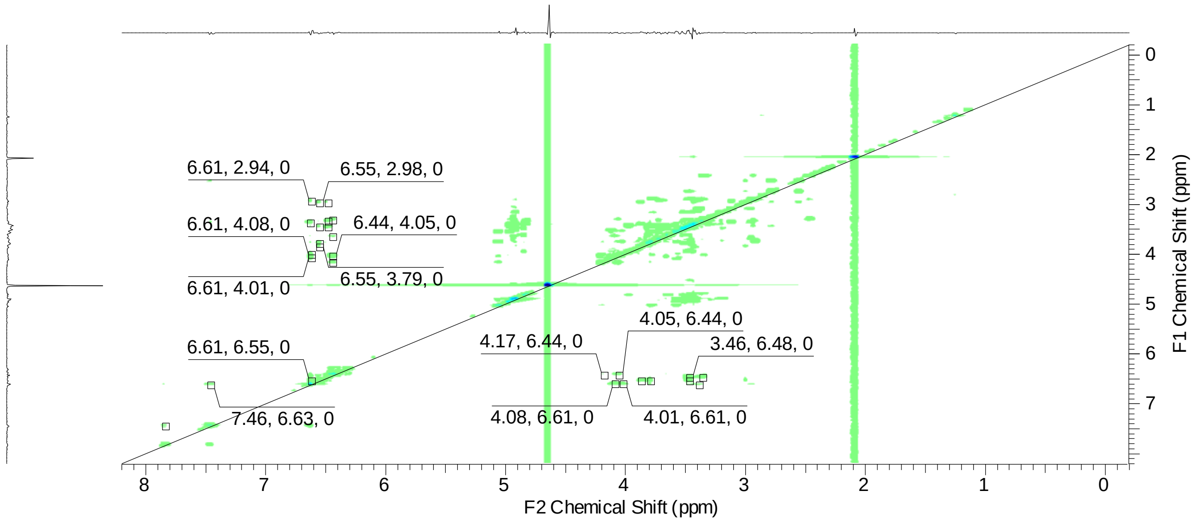

Figure S33

Flu-β-CD

2D ROESY Spectrum

(expansion with peak picking)

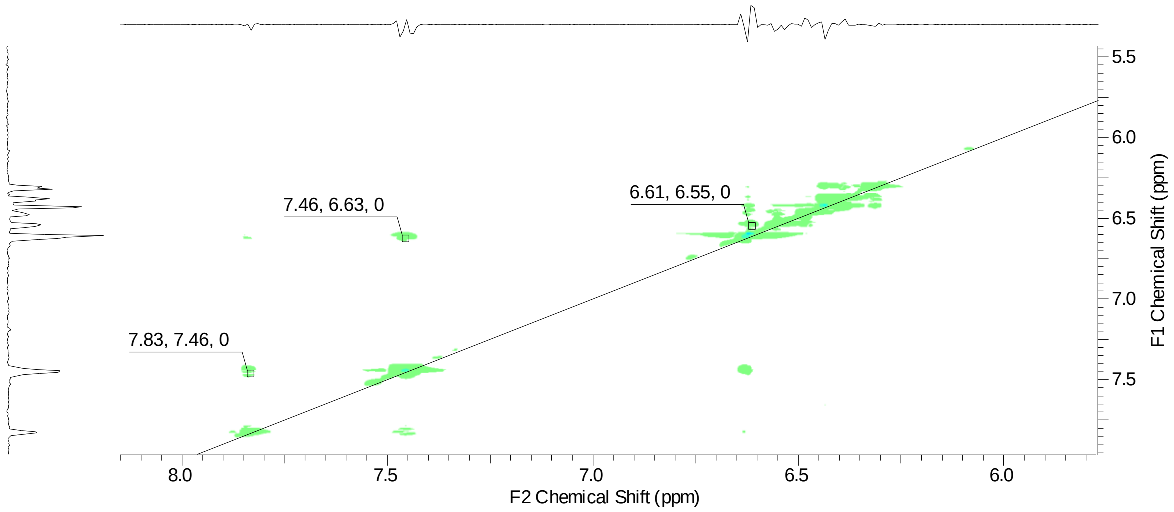

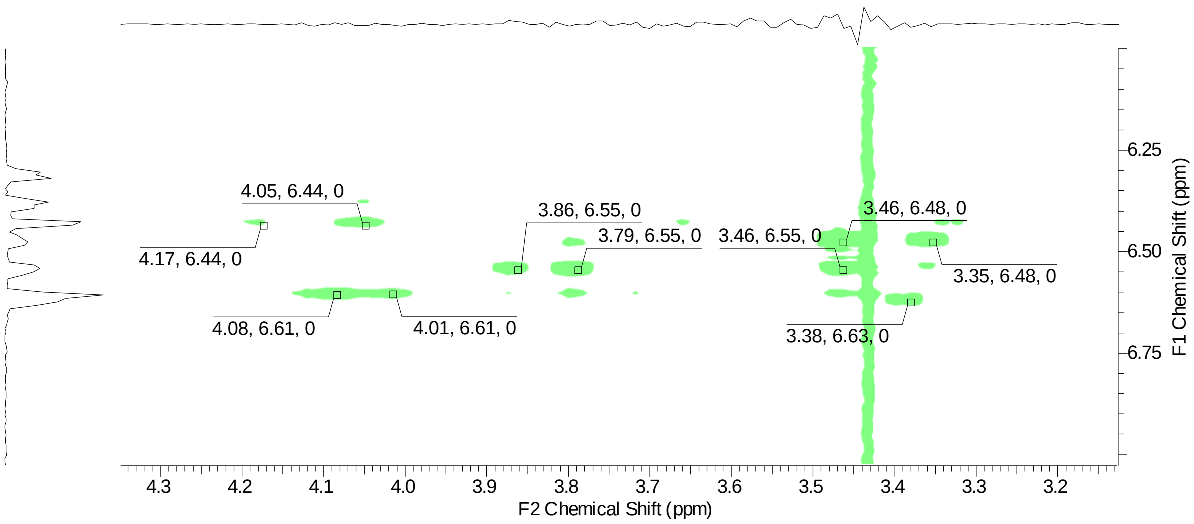

Figure S34

Flu-β-CD

DEPT-ed HSQC Spectrum

(expansion with peak picking)

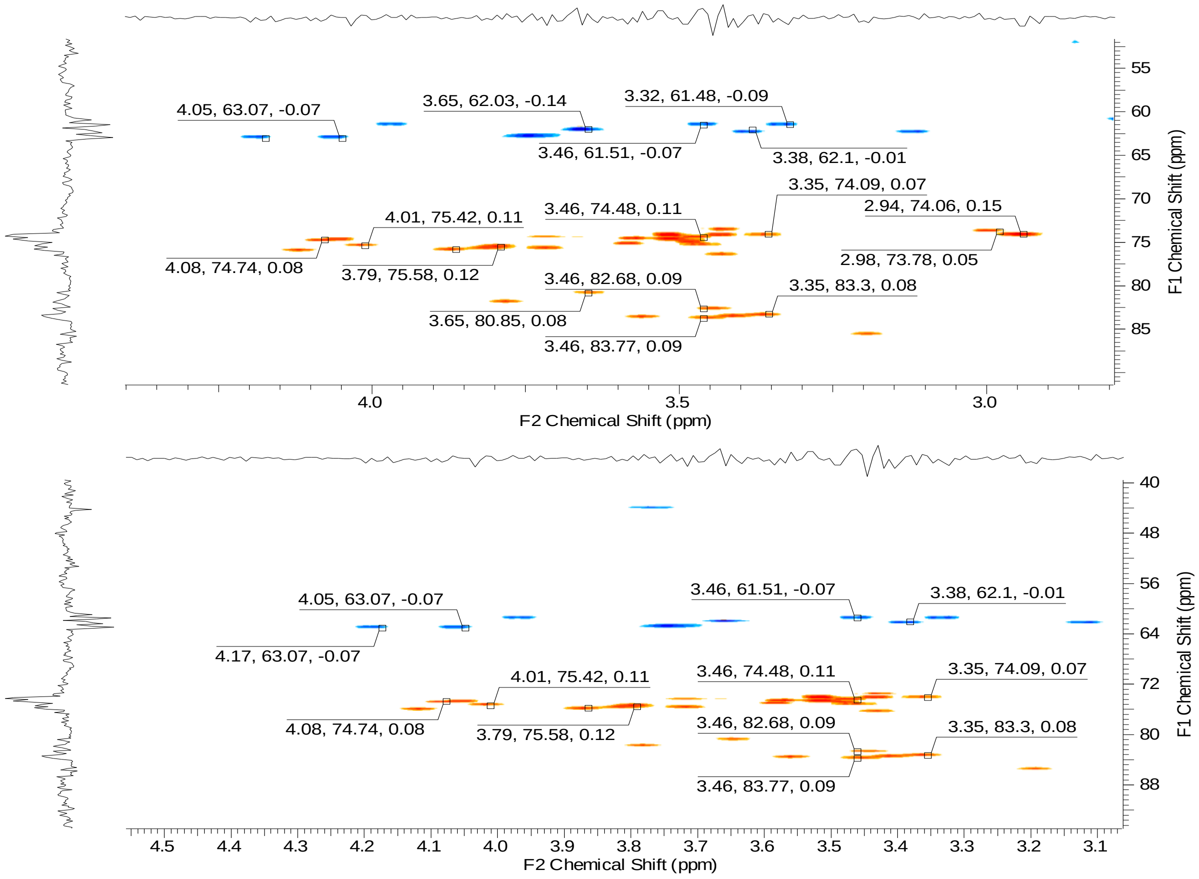

Figure S35

Flu-β-CD

DEPT-ed HSQC Spectrum

(expansion with peak picking)

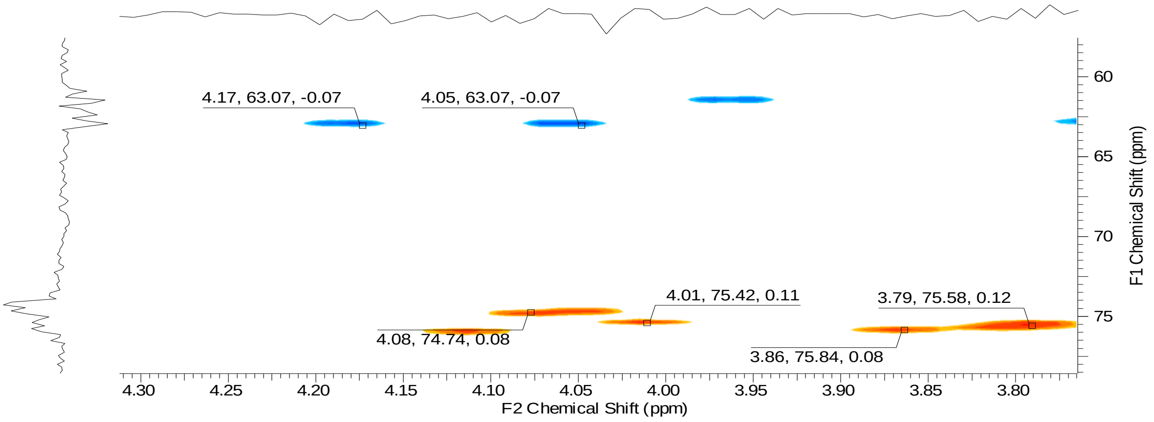

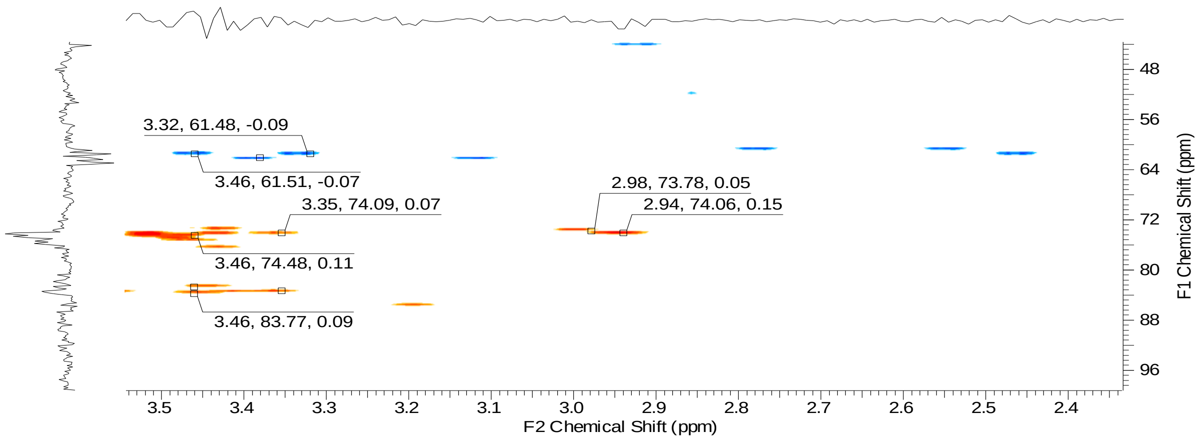

Figure S36

Flu-β-CD

DEPT-ed HSQC Spectrum

(expansion with peak picking)

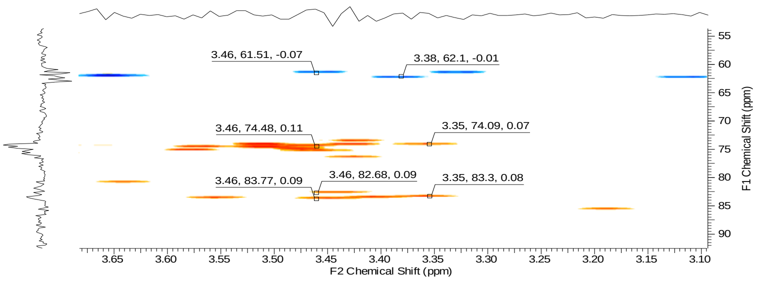

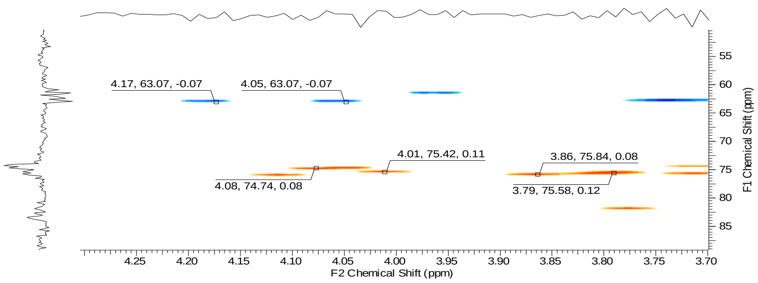

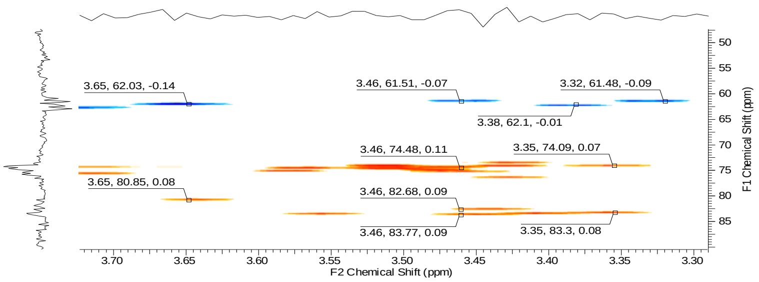

Supplement: File 1 — Experimental section, including IR and NMR spectra of the synthesized compounds. [file Beilstein_J_Org_Chem-12-537-s001.pdf]
